# The transformations of a methylene-bridged bis-triazolium salt: a mesoionic carbene based metallocage and analogues of TCNE and NacNac†‡

**DOI:** 10.1039/d0sc06957d

**Published:** 2021-02-11

**Authors:** Jessica Stubbe, Simon Suhr, Julia Beerhues, Maite Nößler, Biprajit Sarkar

**Affiliations:** Institut Für Chemie und Biochemie, Anorganische Chemie, Freie Universität Berlin Fabeckstraße 34–36 14195 Berlin Germany; Lehrstuhl für Anorganische Koordinationschemie, Institut für Anorganische Chemie, Universität Stuttgart Pfaffenwaldring 55 70569 Stuttgart Germany biprajit.sarkar@iac.uni-stuttgart.de

## Abstract

Unusual and unexpected chemical transformations often provide access to completely new types of functional molecules. We report here the synthesis of a methylene-bridged bis-triazolium salt designed as a precursor for a new bis-mesoionic carbene (MIC) ligand. The direct metalation with silver oxide led to the isolation and crystallographic characterization of a cationic tetranuclear octacarbene–silver(i) complex. During metalation the formal bis-MIC precursor undergoes significant structural changes and chemical transformations. A combined synthetic, crystallographic and (spectro-)electrochemical approach is used to elucidate the mechanistic pathway: starting from the methylene-bridged bis-triazolium salt a single deprotonation leads to a NacNac analogue, which is followed by a redox-induced radical dimerization reaction, generating a new tetra-MIC ligand coordinated to silver(i) central atoms. Decomplexation led to the isolation of the corresponding tetratriazoliumethylene, a profoundly electron-poor alkene, which is an analogue of TCNE.

## Abstract 2

Intriguing chemical transformations are observed for a methylene-bridged bis-triazolium salt leading to a series of interesting functional molecules.

## Graphical abstract

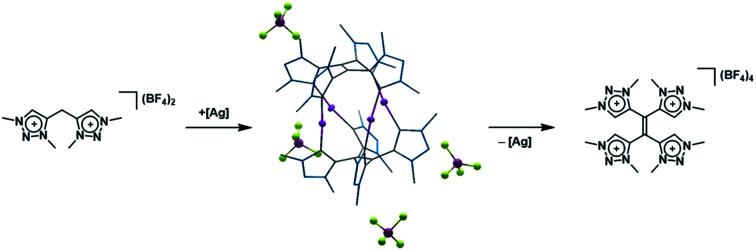

## Introduction

**Fig. 1:**
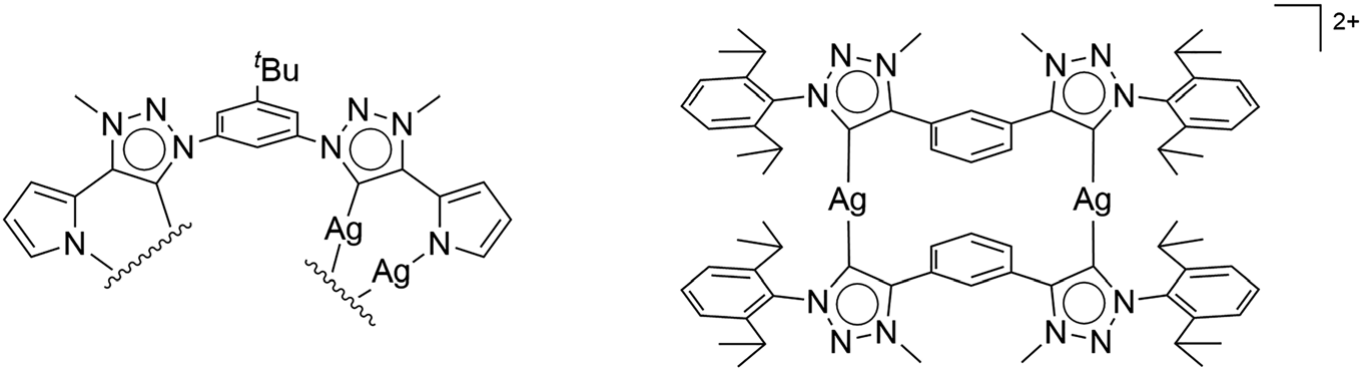
First isolated and crystallographically characterized Ag(i)–MIC complexes by (left) Bielawski and Sessler,^7^ and (right) Crudden.^8^

Due to their efficacy in improving catalyst activities, N-heterocyclic carbenes (NHCs) are among the most important ligand classes in organometallic chemistry.^1^ The popularity of these carbenes predominantly arose because of their strong donor abilities and high covalent bonding to the metal centers.^2^ The majority of publications around NHCs is based on Arduengo-type imidazole-2-ylidenes and 1,2,4-triazol-5-ylidenes,^3^ due to an easy handling of the free carbenes, which are stabilized by heteroatoms adjacent to the carbene.^3*a*^ Alternatively, abnormal NHCs,^4^ especially mesoionic carbenes (MIC) based on 1,2,3-triazol-5-ylidenes,^5^ have been postulated as even better sigma donors.^4*e*^ The corresponding triazoles can be easily constructed in a modular fashion using the copper-catalyzed azide–alkyne cycloaddition, arguably the most famous “click reaction”.^4*d*,6^ The flexibility of the substitution in 1- and 4-position at the triazole is nearly limitless due to the accessibility of a large variety of azides and acetylenes. Either deprotonation of the triazolium salt with a base, or its reaction with Ag_2_O followed by (trans)metalation are typical synthetic routes for generating metal complexes of 1,2,3-triazol-5-ylidenes. Examples of multinuclear silver complexes include the dinuclear Ag(i) complexes reported by Bielawski, Sessler and co-workers^7^ and by Crudden and co-workers^8^ (see Fig. 1). In this context, it should be mentioned that NHCs have been extensively used in the last years in developing metal-based supramolecular assemblies.^9^ Apart from displaying remarkable structures, such supramolecular assemblies have also been used in host–guest recognition chemistry, and for reactions inside confined spaces. The aforementioned silver(i)–triazolylidene complexes constitute structurally characterized intermediates of the popular transmetalation route towards 1,2,3-triazol-5-ylidene-containing complexes. Usually, silver(i)–triazolylidene complexes are unstable and challenging to isolate.^2*a*^ Therefore, the silver often only serves as an auxiliary agent for further complexation, in which the *in situ* generation of the silver–carbene complex is commonly monitored *via* multinuclear NMR spectroscopy.

Here, we report the synthesis of a methylene-bridged, methyl-substituted bis-triazolium salt H_2_**2**(BF_4_)_2_, originally designed as a precursor for the formation of a bis-MIC ligand. In the following, we show the various chemical transformations that we observed while attempting the metalation, or the deprotonation of this salt, and we provide a consistent mechanistic picture for the observations. After metalation with silver oxide, a cationic tetranuclear octacarbene–silver(i) complex could be isolated and crystallographically characterized, which revealed significant structural changes and chemical transformations in the ligand under the chosen conditions. The formal bis-triazolium salt undergoes a single deprotonation, followed by a redox-induced radical dimerization under formal dihydrogen cleavage, leading to the formation of a tetra-MIC-alkene **4**. Decomplexation led to the isolation of H_4_**4**(BF_4_)_4_ as tetratriazoliumethylene, which shows the potential of bearing a comparable rich electron-transfer chemistry as seen for tetracyanoethylene (TCNE). TCNE and related molecules have functioned either as pure organic electron acceptors, or as radical ligands in multinuclear metal complexes. Both metal-free reduced TCNE, and the corresponding TNCE-radical metal complexes display very interesting magnetic and also optical properties.^10^ In the following, a combined synthetic, crystallographic, spectroscopic, (spectro-)electrochemical and theoretical approach is used to describe the products, intermediates and reaction pathways.

## Results and discussion

**Scheme 1:**
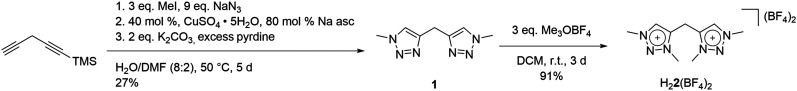
Synthesis of H_2_**2**(BF_4_)_2_.

Our studies began with the synthesis of the bidentate triazole precursor **1**, which was achieved by cycloaddition of methyl azide to the singly TMS-protected 1,4-pentadiyne in the presence of base under standard click conditions (CuSO_4_ and sodium ascorbate) (Scheme 1). The yield is 27%, which is due to safety issues in handling the explosive methyl azide, a reagent which is generated *in situ* and carefully dealt with under diluted conditions, following a synthetic procedure earlier reported from our group.^11^ In proton NMR, the reaction product shows three singlets at 8.23, 4.50 and 4.24 ppm, corresponding to the triazole-H, methylene-bridge and methyl groups, respectively (Fig. S1‡). Methylation with Me_3_OBF_4_ results in the formation of H_2_**2**(BF_4_)_2_ in excellent yields (Scheme 1). A clean bis-methylation was confirmed by the preservation of the protons' chemical equivalency and by the presence of a sharp singlet at 4.24 ppm in proton NMR, corresponding to the methyl groups in the generated triazolium salt (Fig. S3‡).

**Fig. 2:**
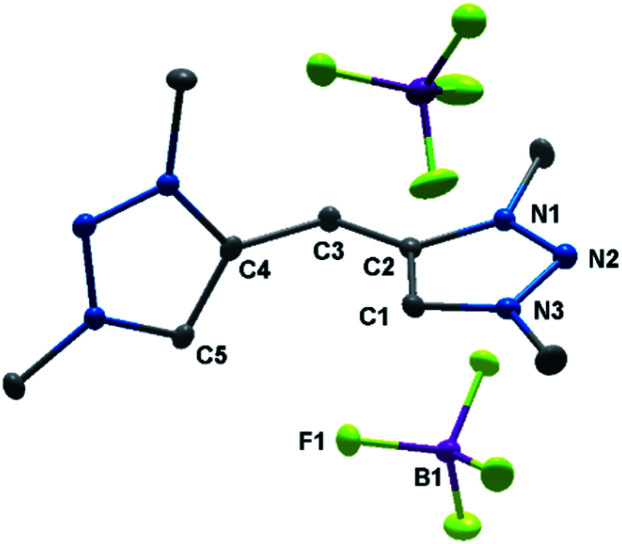
ORTEP representation of H_2_**2**(BF_4_)_2_: ellipsoids drawn at 50% probability. Solvent molecules and H-atoms omitted for clarity (for selected bond length and angles see ESI Table S3‡).

The solid-state molecular structure of H_2_**2**(BF_4_)_2_ displays bond lengths in accordance with values previously reported in the literature (Fig. 2).^5*a*^ The triazole moieties are tilted by 73.2(1)° to each other.

**Scheme 2:**
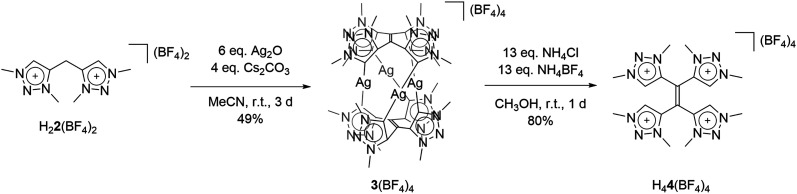
Synthesis of **3**(BF_4_)_4_ and H_4_**4**(BF_4_)_4_.

**Fig. 3:**
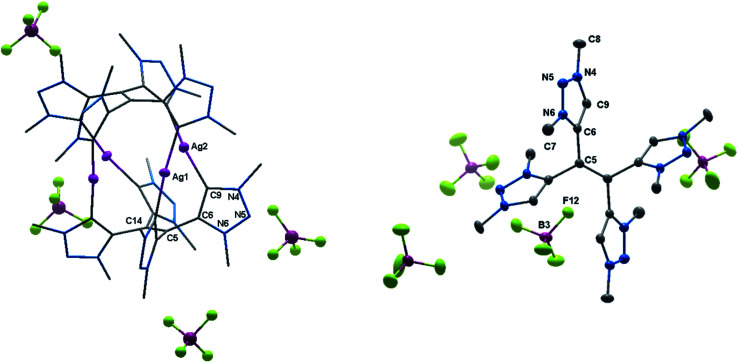
ORTEP representation of (left) **3**(BF_4_)_4_ (for selected bond length and angles see ESI Table S5‡) and (right) H_4_**4**(BF_4_)_4_ (for selected bond length and angles see ESI Table S6‡). Ellipsoids drawn at 50% probability. Solvent molecules and H-atoms omitted for clarity.

With the bis-triazolium precursor in hand, we turned our attention to the coordination chemistry of this ligand. First complexation reactions were accomplished by the direct metalation of H_2_**2**(BF_4_)_2_ with an excess of Ag_2_O in the presence of Cs_2_CO_3_. This procedure is based on standard transmetalation techniques without further treatment of additional metal precursors,^12^ and led to the formation of the air stable tetranuclear silver complex **3**(BF_4_)_4_ (Scheme 2). The molecular structure of **3**(BF_4_)_4_ in the crystal could be unambiguously determined through single crystal X-ray diffraction analysis (Fig. 3, left).

The molecular structure of **3**(BF_4_)_4_ draws attention to significant structural changes and chemical transformations in the native ligand H_2_**2**(BF_4_)_2_ under the metalation conditions: dimerization *via* the formal methylene bridge takes place and leads to the formation of the ethylene-bridged tetra-MIC ligand **4**, in which the newly formed C–C bond (1.336(8) Å) is best described as a double bond. This bond length is shorter compared to C–C bond lengths of tetra-substituted-ethylenes (*ca.* 1.37 Å) which have neutral heteroaromatic substituents.^13^ The reactive nature of the protons in the methylene bridge is also known from methylene-bridged bis-NHC ligands.^14^ However, in many of those cases, the activation of the methylene protons opens up a pathway for decomposition of the compound. The ratio of silver to MIC donors in **3**(BF_4_)_4_ is 1 : 2. Each silver ion is bonded to two MICs and each MIC, four units of which constitute one molecule of ligand **4**, coordinates to one silver(i) center. The overall structure is best described as a sandwich-type metallocage, in which the four silver ions lie in between two ligands of type **4**. However, the structure is not symmetric, as the sandwiching ligands are twisted by 24.7(5)° to each other in order to obtain the nearly linear geometry around the coordinated silver ions. The C_MIC_–Ag–C_MIC_ bond angles range from 172.1(2)° to 176.2(2)°. The average Ag–Ag distance is 3.458(2) Å, and in comparison to the sum of the van der Waals radii for Ag(O) (3.440 Å),^15^ it is consistent with weak metal–metal interactions.^16^ The average Ag–C_MIC_ bond length is 2.086(2) Å and is comparable with previously reported values.^7,8^

In proton NMR, the formation of **3**(BF_4_)_4_ was accompanied by the disappearance of the triazolium-H and the methylene-H_2_ signals (see ESI, S5 and S6‡). A set of four singlets is obtained, corresponding to four inequivalent methyl groups caused by the twisted geometry of **3**(BF_4_)_4_. In ^13^C NMR characteristic resonances at 172.3, 171.4, 170.0 and 168.9 ppm, corresponding to the triazolylidene-C–Ag moieties, are observed.^17^ To the best of our knowledge, this is the first structurally characterized example of a cationic tetranuclear octa-MIC–silver(i) complex. Additionally, the four MICs within the tetra-MIC ligand **4** are connected *via* a C=C double bond, thus delivering a completely new type of a multi-donor MIC ligand.

As **4** was generated *in situ* during the complexation reaction, we were next interested in the decomplexation reaction to generate the corresponding tetratriazolium salt. This was accomplished by the addition of NH_4_Cl to abstract silver as AgCl, and the addition of NH_4_BF_4_ to generate the ligand as the BF_4_^−^ containing tetratriazolium salt H_4_**4**(BF_4_)_4_ (Fig. 3 right, Scheme 2).^18^ In the molecular structure in the crystal, H_4_**4**(BF_4_)_4_ displays a local center of inversion. The central C=C bond length is 1.343(5) Å and fits nicely with a C=C double bond. The C–C and C–N bond lengths within the triazolium units are all in the expected range (Table S5‡).^5*a*^ H_4_**4**(BF_4_)_4_ can thus be described as a tetratriazolium substituted ethylene.

**Fig. 4:**
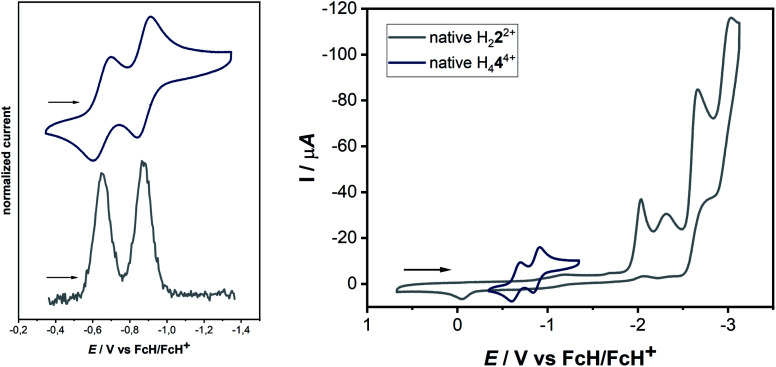
(Left) Cyclic voltammogram and differential pulse voltammogram of a 0.1 mM solution of H_4_**4**(BF_4_)_4_ with 0.1 M NBu_4_PF_6_ in acetonitrile. (Top) 100 mV s^−1^ and (bottom) 20 mV s^−1^ and (right) combined cyclic voltammograms of a 0.1 mM solution of **2**(BF_4_)_2_ and **4**(BF_4_)_4_ with 0.1 M NBu_4_PF_6_ in acetonitrile at 100 mV s^−1^.

**Fig. 5:**
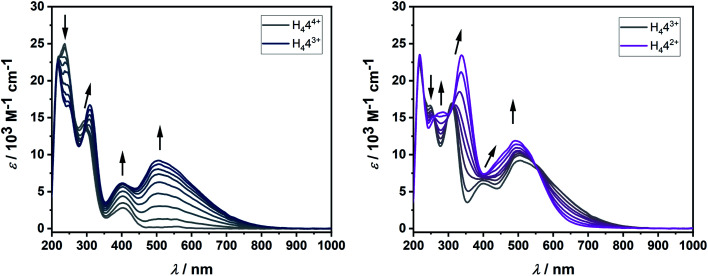
Changes in UV/vis spectrum of H_4_**4**^4+^ in MeCN with 0.1 M NBu_4_PF_6_ during the first reversible reduction (left) and second reversible reduction (right).

In H_4_**4**(BF_4_)_4_ the positively charged triazolium units, which are strongly electron withdrawing groups, induce an electron-poor olefin-moiety, comparable with the famous tetracyanoethylene (TCNE), first reported in 1957 by Middleton and coworkers.^19^ TCNE is a valuable organic compound and finds applications in several different reaction: it is an excellent dienophile, carries good leaving groups, is easy to both oxidize and reduce and is available for coordination to metal centers.^20^ Due to its high electron affinity and reversible reductions, TCNE has been termed the “*E. coli*” of electron-transfer chemistry.^21^ Encouraged by the rich electrochemistry of TCNE, ligand H_4_**4**^4+^ has been investigated by (spectro-)electrochemical methods (Fig. 4, 5 and 8).

The cyclic voltammogram of H_4_**4**(BF_4_)_4_ in CH_3_CN/0.1 M NBu_4_PF_6_ displays two reversible reduction processes at *E*_1/2_ = −0.65 V and *E*_1/2_ = −0.88 V *versus* the ferrocene/ferrocenium couple, with peak-to-peak separations of 95 mV and 69 mV, respectively. The difference in the half-wave potentials is 230 mV, which translates to a comproportionation constant, *K*_c_, value of *ca.* 8 × 10^3^, displaying a reasonable thermodynamic stability of the one-electron reduced form. Further irreversible reductions occur at significantly lower potentials (see ESI, Fig. S10‡). The aforementioned redox-potentials also have similarities to tetrakis(dimethylamino)ethylene (TDAE)^22^ as well as to certain electron-rich olefins derived from carbenes.^23^ However, we believe that a better comparison of H_4_**4**(BF_4_)_4_ is with TCNE (*E*_1/2_(1.Red) = −0.23 V and *E*_1/2_(2.Red) = −0.95 V *vs.* HFc/HFc^+^ in MeCN),^20^ as the tetracationic compound reported here is expected to act as an electron acceptor like TCNE, and not as electron donors like TDAE or the electron-rich olefins. As expected, the tetrasilver(i) complex **3**(BF_4_)_4_ is not very robust towards redox processes. However, in the cyclic voltammogram of **3**(BF_4_)_4_ two distinct reduction steps can be seen at −1.62 and −1.74 V (see ESI, Fig. S16‡). We tentatively assign these redox waves to two two-electron waves that arise from the simultaneous reduction of both the ligands. This would be expected as the two ligands are far apart and do not display either through-space or through-bond electronic coupling. Additionally, the large negative shift of reduction potentials on moving from triazolium salts to the corresponding 1,2,3-triazolylidene-based MICs has been observed earlier.

In solution, the native form of H_4_**4**^4+^ displays absorption bands in the UV region at 219, 234 and 293 nm and in the visible region at 405 nm. Upon one-electron reduction, a broad band appears at 505 nm, while the band at 293 nm is shifted to slightly lower energies. Such low energy bands in the visible or in the NIR region are often observed for organic radicals. The second reduction further shifts this absorption band to 339 nm, while the bands at 405 nm and 505 nm coalesce into one broad band centered at 495 nm. Reversing the potential to the starting potential led to the quantitative regeneration of the spectrum corresponding to the native form H_4_**4**^4+^, thus proving the reversible nature of both the first and second reduction in the UV/vis-NIR spectroelectrochemical time scale.

These observations, including both the electrochemical and spectroelectrochemical behavior, can be compared with those reported for TCNE:^24^ The first reduction potential of H_4_**4**^4+^ is cathodically shifted in comparison to TCNE. Upon one-electron reduction, both compounds display the appearance of a broad band in the visible region (TCNE: ∼420 nm, H_4_**4**^4+^: 505 nm), which decreases upon second reduction for TCNE, but not for H_4_**4**^4+^.

The next question arises regarding the site of reduction in H_4_**4**^4+^. 1,2,3-Triazolium-centered reductions are usually irreversible or in the best case quasi-reversible,^25^ thus it seems unlikely that the reversible reductions observed in the present case are triazolium-centered. This can be elucidated by the direct comparison of the electrochemical behavior of H_4_**4**^4+^ to the native bis-triazolium salt H_2_**2**^2+^ (Fig. 4, right).

The reductions observed for H_2_**2**^2+^ are triazolium-centered, which are at significantly more cathodic potentials and are irreversible. The structural similarities of H_2_**2**^2+^ and H_4_**4**^4+^ allow a careful comparison of the electrochemical behavior, which does not support a triazolium-based reduction in H_4_**4**^4+^. In comparison to TCNE, it seems likely that the electron-poor olefin-moiety of H_4_**4**^4+^ undergoes reversible reductions. Indeed, DFT studies on the singly reduced species support a predominantly olefin-centered reduction.

**Fig. 6:**
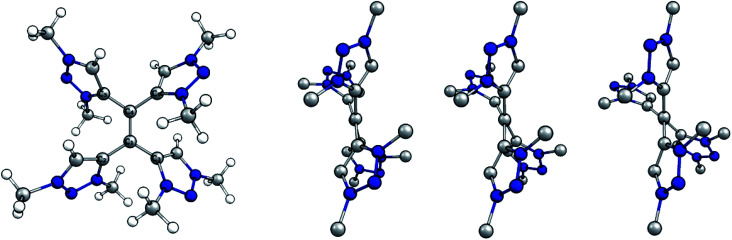
(Left) Optimized structure of native tetracationic species. (Right) Side view along central C–C bond of native, singly and doubly reduced species. H atoms omitted for clarity.

The molecular structures of the native, the singly and the doubly reduced species of [H_4_**4**]^4+^ were optimized at the BP86/def2-TZVP level of theory starting from the crystallographically determined structure H_4_**4**(BF_4_)_4_. The optimized structure of the native species and side views of all calculated species are shown (Fig. 6).

Selected bond distances and dihedral angles are given (Table 1). Two trends are apparent upon reducing the olefinic species: the central C1–C4 bond is elongated while the dihedral angle C2–C1–C4–C6 decreases. Both the increased bond length and the more twisted geometry indicate a decrease in bond order between C1 and C4 upon reduction, which implies the population of π*-orbitals and thus a predominant reduction of the alkene moiety.

**Table 1:** Selected calculated bond distances and dihedral angles

	Native	Singly reduced	Doubly reduced
*r*(C1–C4) [Å]	1.382	1.438	1.479
*r*(C1–C2) [Å]	1.478	1.449	1.422
*d*(C2–C1–C4–C6) [°]	167	152	132

**Fig. 7:**
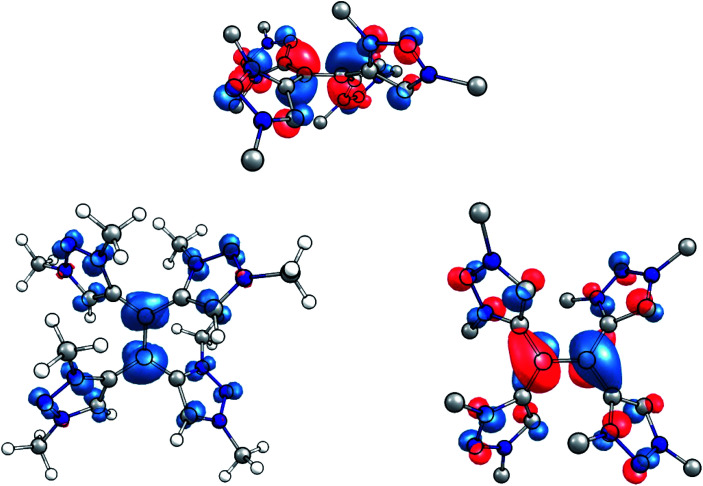
(Left) Spin density of the singly reduced species (contour value 0.005 Å^−1^). (Right) Side and top view of the SOMO of the singly reduced species (contour value 0.05 Å^−1^).

This interpretation is substantiated by an inspection of the spin population in the singly reduced open-shell species, calculated at the TPSSh/def2-TZVP level of theory. According to the Loewdin population analysis, approximately 40% of the spin population are located on the central carbon atoms C1 and C4, while each triazolium-moiety carries about 15% (Fig. 7, left). Furthermore, the SOMO of the one-electron reduced species shows a distinct π*-character with respect to the central C–C bond (Fig. 7, right).

**Fig. 8:**
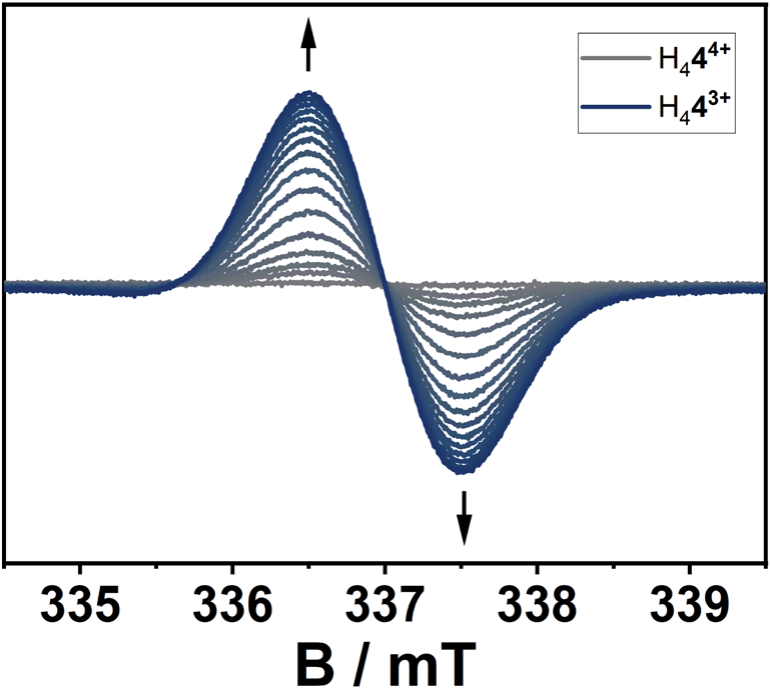
EPR-spectroelectrochemical measurement of H_4_**4**^4+^ with 0.1 M NBu_4_PF_6_ in acetonitrile, electrolysis at −0.2 V *versus* silver wire at 0 °C.

The location of the radical's spin in the singly reduced species was monitored *via* EPR spectroscopy (Fig. 8). Here, the electrochemically generated radical shows a signal centered at *g* = 2.003 without resolved hyperfine coupling to neighboring nuclei, additionally supporting a reduction at the alkene-moiety in significant distance to the nitrogen atoms in the triazolium-moiety. The expected hyperfine couplings are likely not resolved due to unfavorable line-width to hyperfine coupling ratios. A series of simulations using different line widths supports this hypothesis (Fig. S25‡).

According to the combined (spectro-)electrochemical, spectroscopic and theoretical data the reductions can be considered as predominantly olefin-based. The presence of four highly electron withdrawing groups in H_4_**4**^4+^ decreases the electron density around the olefinic group and thus makes it a strong electron acceptor. With suitable electron donors, complexes with intermolecular charge-transfer interactions are expected. In a first attempt at gauging the electron acceptor properties of H_4_**4**^4+^ we reacted it with TDAE (oxidation potentials of −1.01 and −1.18 V *vs.* FcH/FcH^+^ in CH_3_CN). As can be seen from Fig. S21,‡ this reaction leads to a large increase in the signal corresponding to a (TDAE)˙^+^ radical cation, thus displaying successful electron transfer from TDAE to H_4_**4**^4+^. The signal corresponding to H_4_**4**^3+^˙, which is expected to be featureless (Fig. 8), is likely “hidden” below the intense and well-resolved signal of (TDAE)˙^+^. Further detailed studies will be necessary to determine the exact nature of the compound that is formed.

**Fig. 9:**
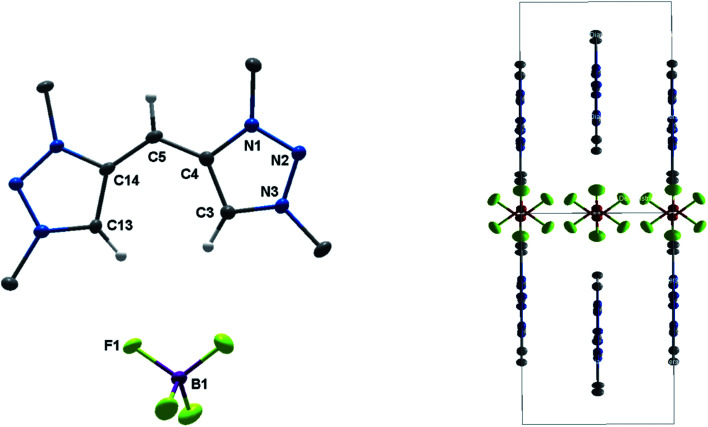
ORTEP representation of [H_2_**2**^2+^–H^+^](BF_4_): ellipsoids drawn at 50% probability. H-atoms (except those at C3, C5 and C13) are omitted for clarity. (Left) Single molecule, (right) unit cell (for selected bond length and angles see ESI Table S4‡).

Eventually, the structural changes from H_2_**2**(BF_4_)_2_ to H_4_**4**(BF_4_)_4_ within the metalation is still an open question. At first, we focused on the deprotonation of H_2_**2**(BF_4_)_2_, in which we were able to isolate the singly deprotonated species [H_2_**2**^2+^–H^+^](BF_4_) (Fig. 9).

**Scheme 3:**
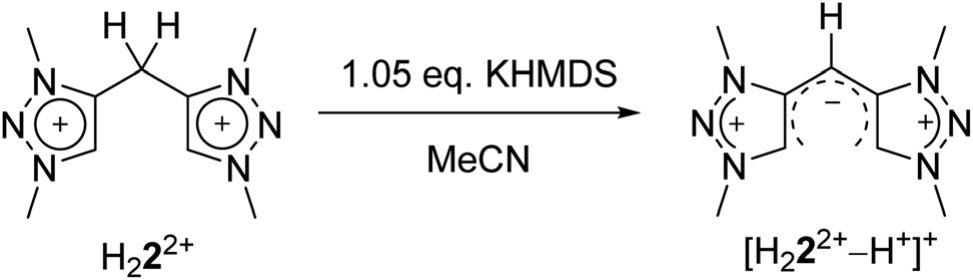
Synthesis of [H_2_**2**^2+^–H^+^]^+^.

In the solid state, the molecule is perfectly planar and nearly symmetric. Slight discrepancies in symmetry are caused by different bond lengths between the bridging C–H moiety and the triazolium units with 1.404(5) Å and 1.388(6) Å. However, both bond lengths are shorter in comparison to those observed in H_2_**2**(BF_4_)_2_, which give them considerable double bond character. In order to maintain the symmetry and planarity, the charges in this molecule appear to be separated, yet delocalized (Scheme 3).

**Scheme 4:**
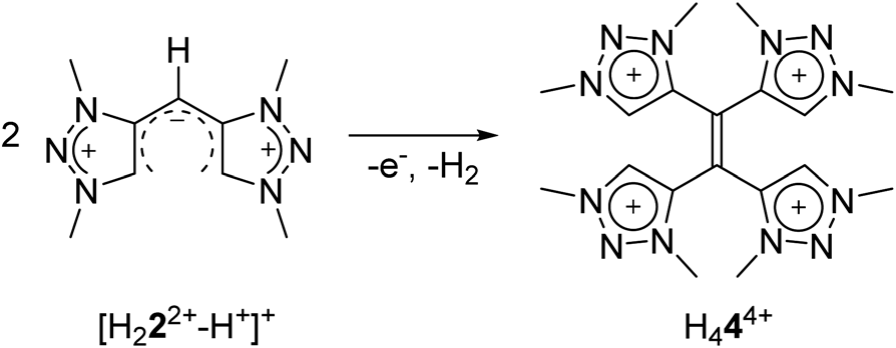
Formation of H_4_**4**^4+^ from [H_2_**2**^2+^–H^+^]^+^.

**Fig. 10:**
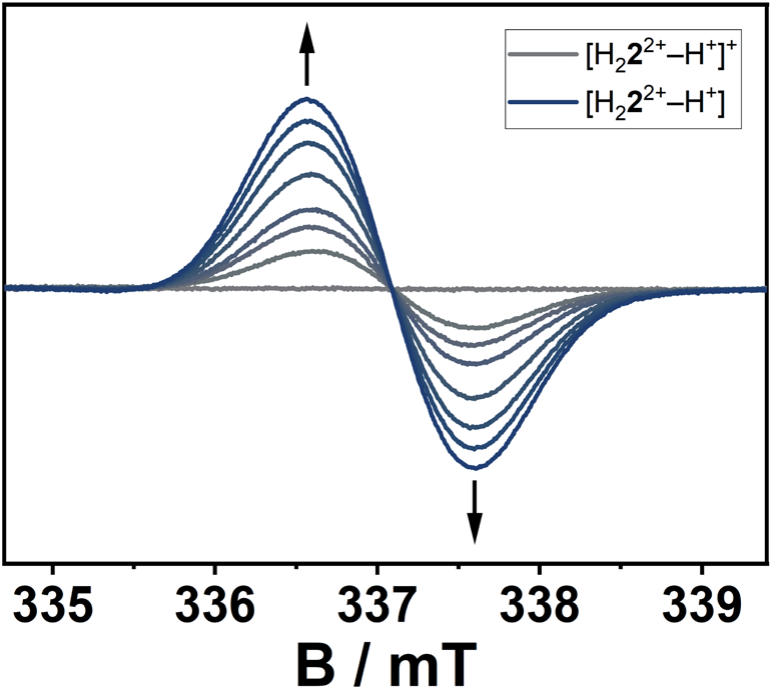
EPR-spectroelectrochemical measurement of [H_2_**2**^2+^–H^+^]^+^ with 0.1 M NBu_4_PF_6_ in acetonitrile, electrolysis at 0.8 V *versus* silver wire at 0 °C.

The conversion of H_2_**2**(BF_4_)_2_ to [H_2_**2**^2+^–H^+^](BF_4_) is supported by NMR spectroscopic and single crystal X-ray diffraction measurements (see ESI, Fig. S3 and S9,‡ and above). The rearrangement is then followed by a redox-induced radical dimerization under formal dihydrogen cleavage (Scheme 4): oxidation of [H_2_**2**^2+^–H^+^]^+^ at the methylene-bridging carbon leads to the formation of an open-shell species, which we were able to generate electrochemically after the *in situ* deprotonation of H_2_**2**(BF_4_)_2_. The open-shell species was monitored *via* EPR spectroscopy. The resulting spectrum does not show any hyperfine coupling to neighboring nuclei and is centered at *g* = 2.004 (Fig. 10).

**Fig. 11:**
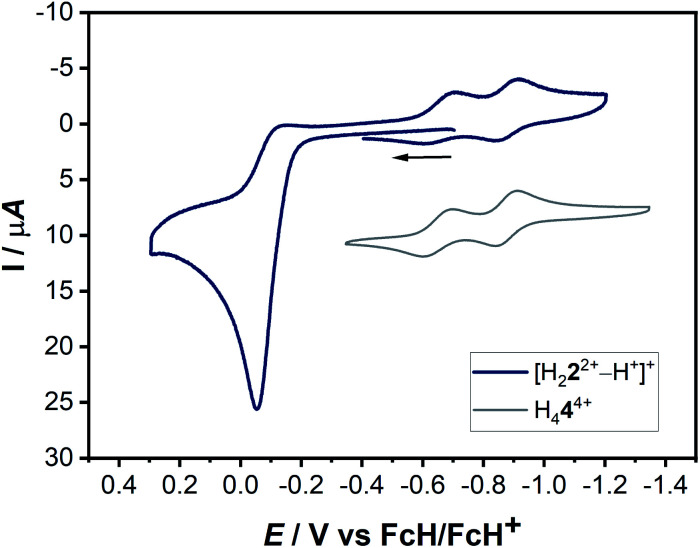
Combined cyclic voltammograms of a 0.1 mM solution of [H_2_**2**^2+^–H^+^]^+^ and H_4_**4**^4+^ with 0.1 M NBu_4_PF_6_ in acetonitrile at 100 mV s^−1^.

The actual formation of H_4_**4**(BF_4_)_4_ through radical dimerization (Scheme 4) was confirmed *via* cyclic voltammetry (Fig. 11).

Here, the *in situ* generated [H_2_**2**^2+^–H^+^]^+^ initially displays an oxidation step at *ca.* −0.05 V (Fig. 11). On reversing the potential at 0.3 V *versus* the ferrocene/ferrocenium couple, dimerization takes place within the CV timescale, thus leading to the appearance of the characteristic two olefin-centered reduction waves of H_4_**4**^4+^ at *E*_1/2_ = −0.65 V and *E*_1/2_ = −0.88 V, with peak-to-peak separations of 95 mV and 69 mV, respectively. These new reduction peaks corresponding to H_4_**4**^4+^ appear in response to the aforementioned oxidation process. The combination of all these control experiments thus provide insights into the formation of H_4_**4**^4+^.

**Scheme 5:**
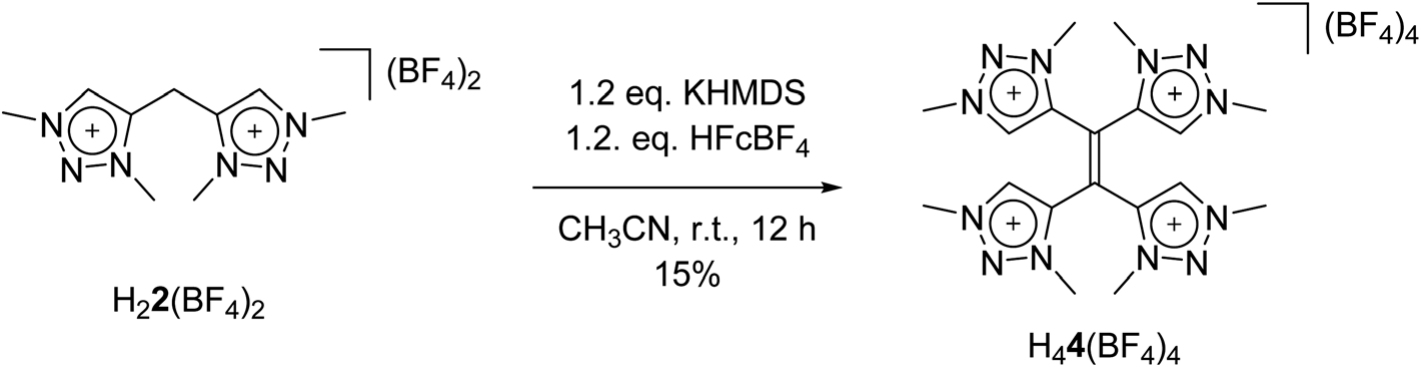
Direct synthesis of H_4_**4**(BF_4_)_4_.

With these observations in hand, we set out to decipher the conditions under which H_4_**4**(BF_4_)_4_ can be directly synthesized starting from H_2_**2**(BF_4_)_2_ (Scheme 5). We investigated the deprotonation reaction with several bases, in which KHMDS proved to be an appropriate base for a clean single deprotonation (see ESI, Fig. S9‡). The oxidation requires a mild oxidizing agent such as ferrocenium tetrafluoroborate. The reaction leads to a mixture of starting material and product, in which the desired tetratriazoliumethylene H_4_**4**(BF_4_)_4_ can be isolated after recrystallization, and the starting material regained. Attempts for the optimization of this reaction are currently being pursued in our group. The formation of H_4_**4**(BF_4_)_4_ through the activation of the methylene protons of H_2_**2**(BF_4_)_2_ as described here is reminiscent of a few recent investigations on the transformations of bis-heteroaromatic substituted methanide ligands.^26^

In conclusion, we have presented the synthesis of a new bis-triazolium salt, which undergoes significant structural changes and chemical transformations under metalation conditions with silver oxide. We were able to isolate and crystallographically characterize the cationic tetranuclear octacarbene–silver(i) metallocage and the corresponding newly formed tetratriazoliumethylene through decomplexation. An independent synthesis of the tetratriazoliumehtylene has been presented as well which does not require the prior formation of the silver-based metallocage. Here, we could reveal important aspects of the mechanistic pathways: a single deprotonation, followed by a redox-induced radical dimerization causes the structural changes and chemical transformations from the formal bis-triazolium salt to the tetratriazoliumethylene. The newly formed tetratriazolium alkene is an analogue of TCNE, which could be illustrated by the first (spectro-)electrochemical investigations on that compound. Furthermore, the electron acceptor ability of the tetratriazoliumethylene was proven by its reduction with the electron donor TDAE. Additionally, the singly deprotonated compound (a NacNac analogue) and the tetratriazolium salt are expected to be exciting ligands for generating a host of functional metal complexes. The compounds reported here are thus likely to be relevant for diverse fields of chemistry such as organic materials, redox-active supramolecular cages, and redox-switchable catalysis.

## Conflicts of interest

There are no conflicts to declare.

## Supplementary Material

SC-012-D0SC06957D-s001

SC-012-D0SC06957D-s002
