# Supplementary material for: The transformations of a methylene-bridged bis-triazolium salt: a mesoionic carbene based metallocage and analogues of TCNE and NacNac
Source: Chem Sci. 2021 Feb 11;12(9):3170–8. doi: 10.1039/d0sc06957d (PMC8179415; doi:10.1039/d0sc06957d)
Supplement: SC-012-D0SC06957D-s001 [file SC-012-D0SC06957D-s001.pdf]

Electronic Supplementary Material (ESI) for Chemical Science.
This journal is © The Royal Society of Chemistry 2021

Supporting Information

1. Instrumentation 2
2. Synthetic Procedures 4
1. NMR spectra 9
2. Cyclic Voltammetry 14
3. Spectroelectrochemistry 17
4. EPR-monitored treatment of $[H_4\mathbf{4}]^{4+}$ with Tetrakis(dimethylamino)ethylene (TDAE) 19
5. DFT 20
6. Crystal Structures & crystallographic data 29
7. References 34

1. Instrumentation

Unless otherwise noted, all reactions were performed using standard Schlenk-line techniques under an inter atmosphere of argon (Linde, Argon 4.8, purity ≥ 99.998). Commercially available chemicals were used without further purification. THF, diethyl ether and *n*-hexane were dried and distilled from sodium, dichloromethane and acetonitrile from phosphorus pentoxide. Other solvents were available from MBRAUN MB-SPS-800 solvent system and additionally degassed using standard techniques.

^{1}H NMR were recorded on Joel ECS 400, JOEL 400R, Fa. BRUKER AVANCE500 or AVANCE700 proton resonance spectrometer. Proton decoupled ^{13}C NMR were recorded on Fa. BRUKER AVANCE700 proton resonance spectrometer. Chemical shifts are reported in ppm (relative to the TMS signal) with reference to the residual solvent peaks.[1] Multiplets are reported as follows: singlet (s), duplet (d), triplet (t), quartet (q), quintet (quint), and combinations thereof.

Electrochemistry

Cyclic voltammograms were recorded with a PAR VersaStat 4 potentiostat (Ametek) by working in anhydrous and degassed acetonitrile with 0.1 M NBu_4PF_6 (dried, > 99.0%, electrochemical grade, Fluka) as supporting electrolyte. Concentrations of the compounds were about $1\cdot10^{-4}$ M. A three-electrode setup was used with a glassy carbon working electrode, a coiled platinum wire as counter electrode, and a coiled silver wire as a pseudo-reference electrode. The ferrocene/ferrocenium couple was used as internal reference.

Spectroelectrochemistry

UV/Vis spectra were recorded with an Avantes spectrometer consisting of a light source (AvaLight-DH-S-Bal), a UV/vis detector (AcaSpec-ULS2048), and an NIR detector (AvaSpec-NIR256-TEC). Spectroelectrochemical measurements were carried out in an optically transparent thin-layer electrochemical (OTTLE) cell (CaF_2 windows) with a gold working electrode, a platinum mesh counter electrode, and a silver-foil pseudoreference electrode.[2] Anhydrous and degassed acetonitrile with 0.1 M NBu_4PF_6 as electrolyte was used as solvent.

EPR spectra at X-band frequency (ca. 9.5 GHz) were obtained with a Magnettech MS-5000 benchtop EPR spectrometer equipped with a rectangular TE 102 cavity and TC HO4 temperature controller. The measurements were performed in synthetic quartz glass tubes. For

Electron Paramagnetic Resonance

EPR spectroelectrochemistry a three-electrode setup was employed using two Teflon-coated platinum wires (0.005 in. bare, 0.008 in. coated) as working and counter electrodes and a Teflon-coated silver wire (0.005 in. bare, 0.007 in. coated) as pseudoreference electrode. Spectral simulations were performed with EasySpin 5.1.4.[3] and MatLab R2012a.

Theoretical approach

All calculations were performed using the ORCA software package, versions 4.2.0 or 4.2.1.[4-5] Starting from the crystallographically determined structure of H_4**4**$^{4+}$, geometry optimizations were performed in

the gas phase using the BP86/def2-TZVP level of theory.[6-7] Minima were verified by absence of imaginary frequencies in analytical frequency calculations. Dispersive interactions were accounted for by using Grimme's D3 approach with Becke-Johnson damping (D3-BJ).[8-11] Single point calculations were performed on minimum geometries using a def2-TZVPP basis set and the following functionals: TPSSh[12] (hybrid meta-GGA), TPSS[12](meta-GGA), B3LYP[13] (hybrid GGA) and M06-2X[14] (hybrid meta-GGA). RI or RIJCOSX approximations with appropriate auxiliary basis sets were employed in all calculations to reduce computational time.[15-22]

Single-Crystal X-Ray Diffraction

X-ray data were collected on a Bruker Smart AXS or Bruker D8 Venture systems at 140(2) K or 100(2) K, respectively, using graphite-monochromated $Mo_{K\alpha}$ radiation (λ_{α}= 0.71073 Å). The strategy for the data collection was evaluated by using the Smart software. The data were collected by the standard omega scan or omega + phi scan techniques, and were scaled and reduced using Saint+ and SADABS software. The structures were solved by direct methods using SHELXS-97 or intrinsic phasing using SHELXL-2014/7 and refined by full matrix least-squares, refining on F^2. Non-hydrogen atoms were refined anisotropically.[23-28]

2. Synthetic Procedures

The synthesis was done by a modified literature procedure.[29]

Attention: Methyl azide is highly explosive and should be handled with great care. Although we never experienced any problems, the reaction should only be attempted in high dilutions.

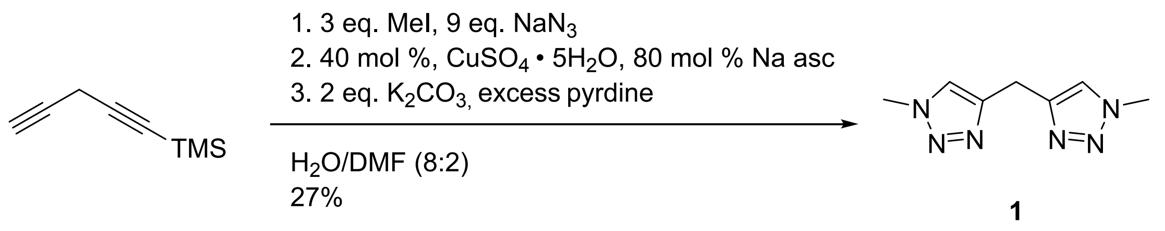

Under ambient conditions sodium azide (2.15 g, 33.0 mmol) and methyl iodide (0.69 mL, 11.0 mmol) were stirred in a water (40 mL) / dimethylformamide (10 mL) mixture at 50 °C for 24 h. The mixture was cooled to room temperature and 1-trimethylsilyl-1,4-pentadiyn (0.64 mL, 3.7 mmol), copper sulfate pentahydrate (0.37 g, 1.5 mmol), sodium ascorbate (0.58 g, 3.0 mmol), potassium carbonate (1.01 g, 7.3 mmol) and pyridine (2.5 mL) was added. The suspension was warmed to 50 °C and stirred for additional 5 days.

The mixture was allowed to cool to room temperature, quenched with aqueous Na_2EDTA/NH_4OH solution and extracted with dichloromethane. The combined organic layers were dried with Na_2SO_4. Removal of the volatiles gave a brown oil, which was dissolved in small amounts of dichloromethane and added into an excess of *n*-hexane. The precipitant was collected by filtration as a grey solid and dried *in vacuo*. The crude product was purified by sublimation (10^{-3} mbar at 110 °C) to give a white solid in 27% yields (174.0 mg).

^{1}H NMR (400 MHz, $CDCl_3$, 20 °C): δ = 7.44 (s, 2H, triazole-*H*), 4.19 (s, 2H, CH_2), 4.05 (s, 6H, CH_3) ppm.

^{13}C NMR (101 MHz, $CDCl_3$, 22 °C): δ = 145.8 (C=*C*–C), 122.9 (*C*H), 36.8 (*C*H_3), 22.9 (*C*H_2) ppm.

Elemental analysis calcd. (%) for $C_7H_{10}N_6$: C 47.18, H 5.66, N 47.16; found: C 47.97, H 5.70, N 47.36.

$H_2\mathbf{2}(BF_4)_2$

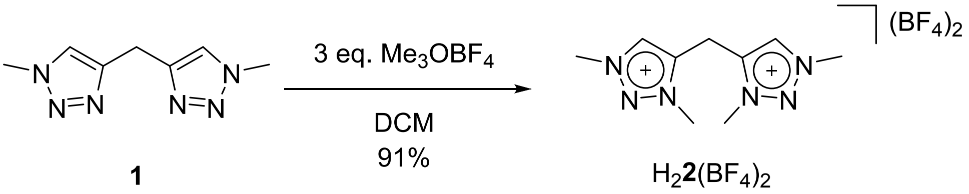

Under an inert atmosphere of argon **1** (119.4 mg, 0.67 mmol) and trimethyloxonium tetrafluoroborate (297.3 mg, 2.01 mmol) were dissolved in dry dichloro-methane (20 mL). The mixture was stirred for 3 d at room temperature. The solution was quenched with methanol (5 mL) and the mixture was added to diethyl ether (100 mL) under ambient conditions. The white precipitant was collected by filtration, suspended in dichloromethane and kept for 30 min in an ultrasonic bath. The solid was collected by filtration, washed with dichloromethane and dried *in vacuo*. The product was obtained as white solid in very good yields of 91% (232.8 mg).

^{1}H NMR (500 MHz, CD_3CN, 27 °C): δ 8.23 (s, 2H, triazolium-*H*), 4.50 (s, 2H, CH_2), 4.24 (s, 6H, CH_3), 4.18 (s, 6H, CH_3) ppm.

^{13}C NMR (126 MHz, CD_3CN, 27 °C): δ 255.2 (C=*C*–C), 248.7 (C*H*), 158.2(*C*H_3), 156.0 (*C*H_3), 136.1 (*C*H_2) ppm.

Elemental analysis calcd. (%) $C_9H_{16}B_2F_8N_6$: C 28.31, H 4.22, N 22.01; found: C 28.84, H 4.32, N 22.79.

$[H_2\mathbf{2}^{2+}–H^+](BF_4)$

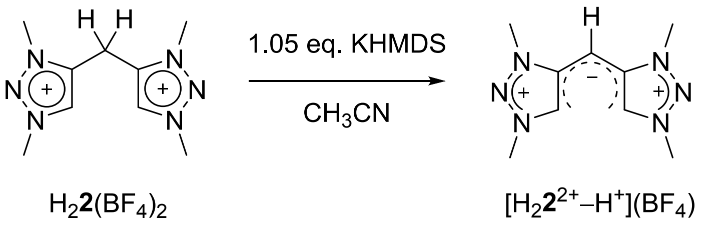

Under an inert atmosphere of argon $H_2\mathbf{2}(BF_4)_2$ (50.0 mg, 0.131 mmol) and KHMDS (27.4 mg, 0.137 mmol) were dissolved in dry CD_3CN (3 mL). The mixture was stirred for 2 hours. Single crystals of $[H_2\mathbf{2}^{2+}–H^+](BF_4)$ could be obtained by the slow diffusion of diethyl ether to the solution under inert conditions. $[H_2\mathbf{2}^{2+}–H^+](BF_4)$ could not be isolated in the pure form as bulk material.

NMR Experiment: Under an inert atmosphere of argon $H_2\mathbf{2}(BF_4)_2$ (6.9 mg, 0.018 mmol) and KHMDS (3.8 mg, 0.019 mmol) were dissolved in dry and degassed CD_3CN (1 mL).

3$(BF_4)_4$

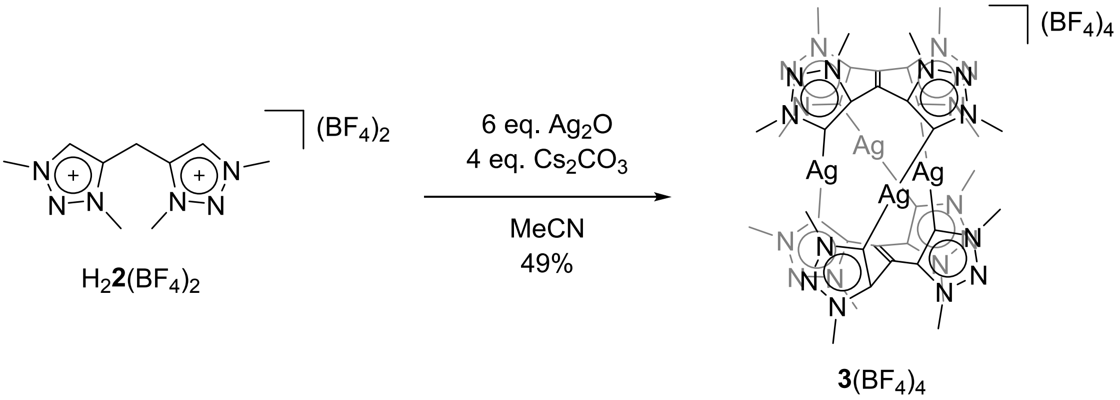

Under an inert atmosphere of argon H_2**2**$(BF_4)_2$ (50.0 mg, 0.131 mmol), freshly prepared silver oxide (182.2 mg, 0.786 mmol) and cesium carbonate (128.1 mg, 0.393 mmol) were dissolved in dry acetonitrile (10 mL). The suspension was stirred for 3 d under the exclusion of light. The mixture was filtered through a syringe filter (PTFE, 0.2 µm) and volatiles were removed under reduced pressure. The crude brown oil was purified by fractional crystallization: The oil was dissolved in small amounts of acetonitrile and diethyl ether was added by slow diffusion. During this process a brown precipitant was obtained and removed. The process was repeated until no brown precipitant was obtained anymore and the product crystallized as yellow single crystals in 49 % yields (25.6 mg).

^{1}H NMR (700 MHz, CD_3CN, 27 °C): δ 4.13 (s, 12H) 4.00 (s, 12H) 3.91 (s, 12H) 3.82 (s, 12H) ppm.

^{13}C NMR (176 MHz, CD_3CN, 27 °C): δ 173.3, 172.3, 170.8, 169.8, 145.4, 143.8, 129.5, 44.4, 43.7, 38.5, 38.1 ppm.

Elemental analysis calcd. (%) $C_{36}H_{48}Ag_4B_4F_{16}N_{24}$: C 27.10, H 3.03, N 21.07; found: C 27.64, H 4.36, N 23.01.

$H_4\mathbf{4}(BF_4)_4$

Method **A**

The synthesis was adapted from the syntheses of analogous imidazolium compounds.[30]

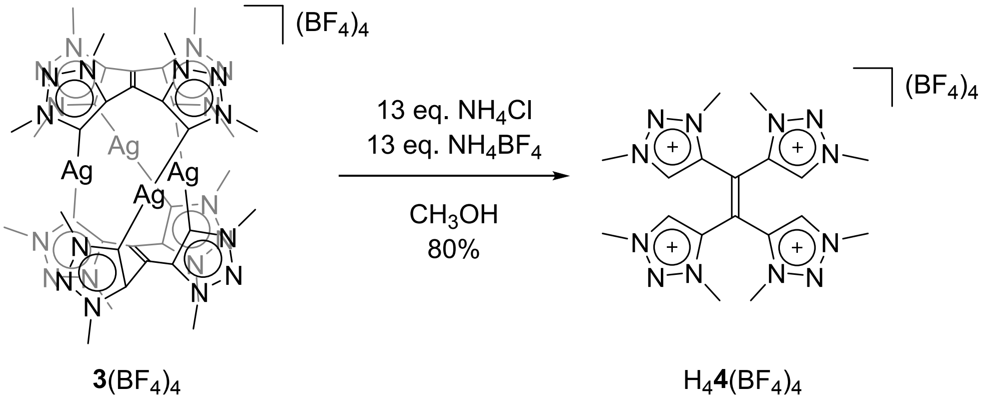

Under ambient conditions $\mathbf{3}(BF_4)_4$ (25.6 mg, 0.016 mmol) was dissolved in methanol (10 mL) and ammonium chloride (11.1 mg, 0.208 mmol) was added. The solution was stirred overnight and the precipitated silver chloride filtered off. Ammonium tetrafluoroborate (25.2 mg, 0.240 mmol) was added and the solution kept stirring for additional 12 h. The white precipitant was filtered, re-dissolved in acetonitrile and filtered again. All volatiles were removed under reduced pressure and the product was obtained as a yellow solid in 80% yields (9.7 mg). Single crystals were obtained by the slow diffusion of diethyl ether in an acetonitrile solution of the product.

Method **B**

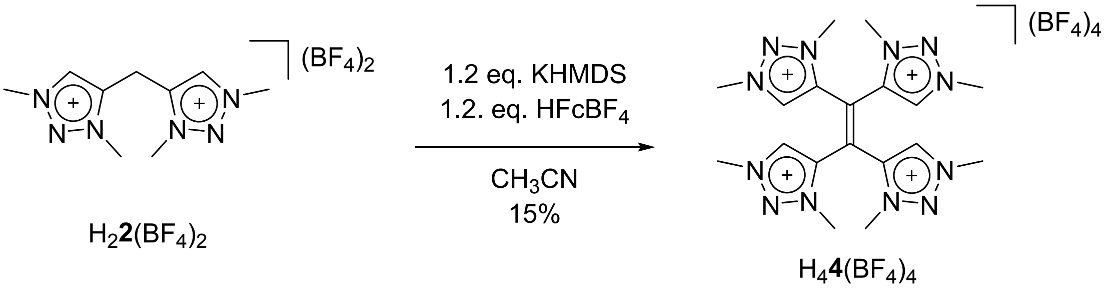

Under an inert atmosphere of argon H_2**2**$(BF_4)_2$ (50.0 mg, 0.131 mmol) was dissolved in dry acetonitrile. Potassium bis(trimethylsilyl)amide (31.3 mg, 0.157 mmol) was added and the solution stirred for 30 min. Ferrocenium tetrafluoroborate (42.8 mg, 0.157 mmol) was added and the solution kept stirring overnight. Solids in the reaction mixture were removed by filtration over celite and the volatiles were removed under reduced pressure. The remaining brown oil was redissolved in dichloromethane, to remove the ferrocene/ferrocenium leftovers by filtration. The solid was redissolved in acetonitrile and added to an excess of diethyl ether. The precipitant was collected, containing a mixture of starting material and product. The product can be separated from the starting material in a pure form by recrystallization in acetonitrile and diethyl ether in 15% (15.2 mg).

^{1}H NMR (700 MHz, CD_3CN, 27 °C): δ 8.66 (s, 4H, triazole-*H*), 4.30 (s, 12H, Me-*H*), 4.10 (s, 12H, Me-*H*) ppm.

^{13}C NMR (176.1 MHz, CD_3CN, 27 °C): δ 137.0 (trz-*C*H), 134.1 (trz-$C_{quart.}$), 124.2 ($C_{quart.}$), 44.7 (CH_3), 41.1 (CH_3) ppm.

Elemental analysis calcd. (%) $C_{18}H_{28}B_4F_{16}N_{12}$: C 28.46, H 3.72, N 22.12; found: C 28.51, H 4.01, N 22.35.

UV/Vis: 218 nm (23700 $M^{-1}\cdot cm^{-1}$), 231 nm (24900 $M^{-1}\cdot cm^{-1}$), 292 nm (14906 $M^{-1}\cdot cm^{-1}$), 401 nm (3000 $M^{-1}\cdot cm^{-1}$).

1. NMR spectra

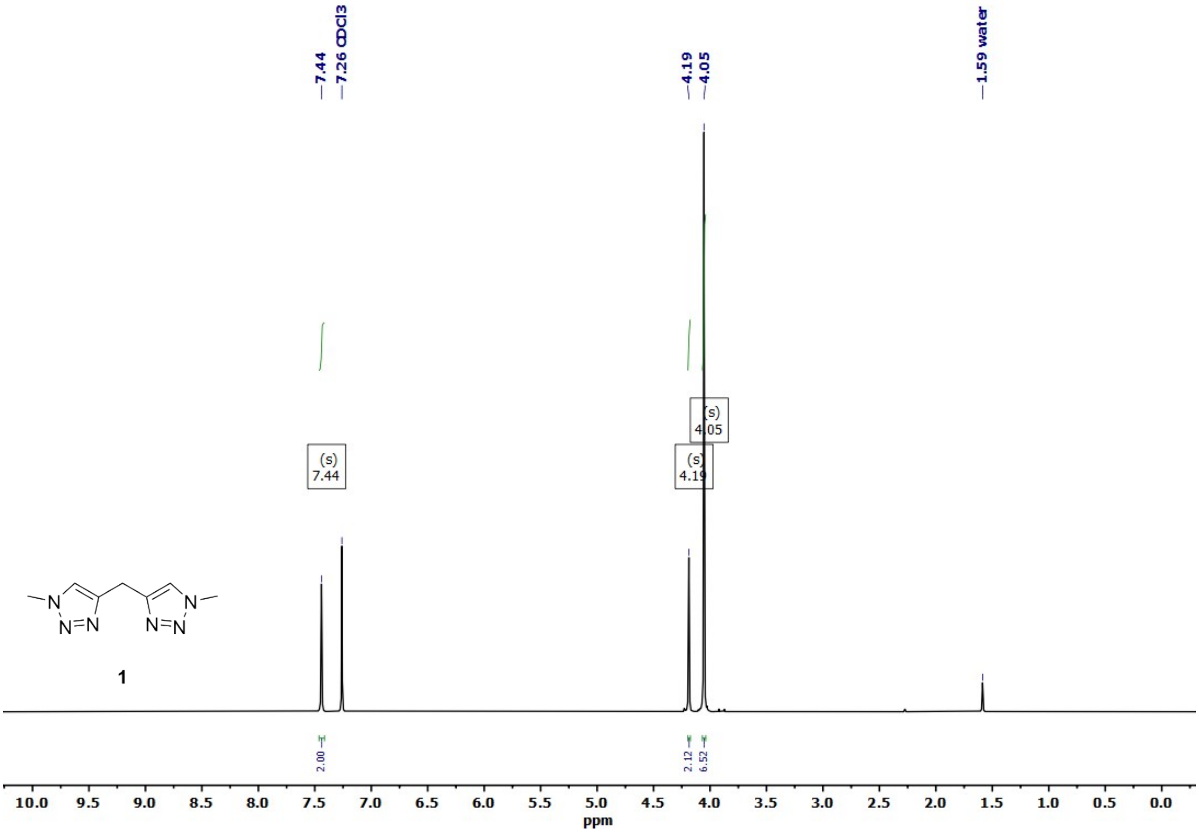

Figure S1: *^{1}H NMR spectra of **1** in $CDCl_3$.*

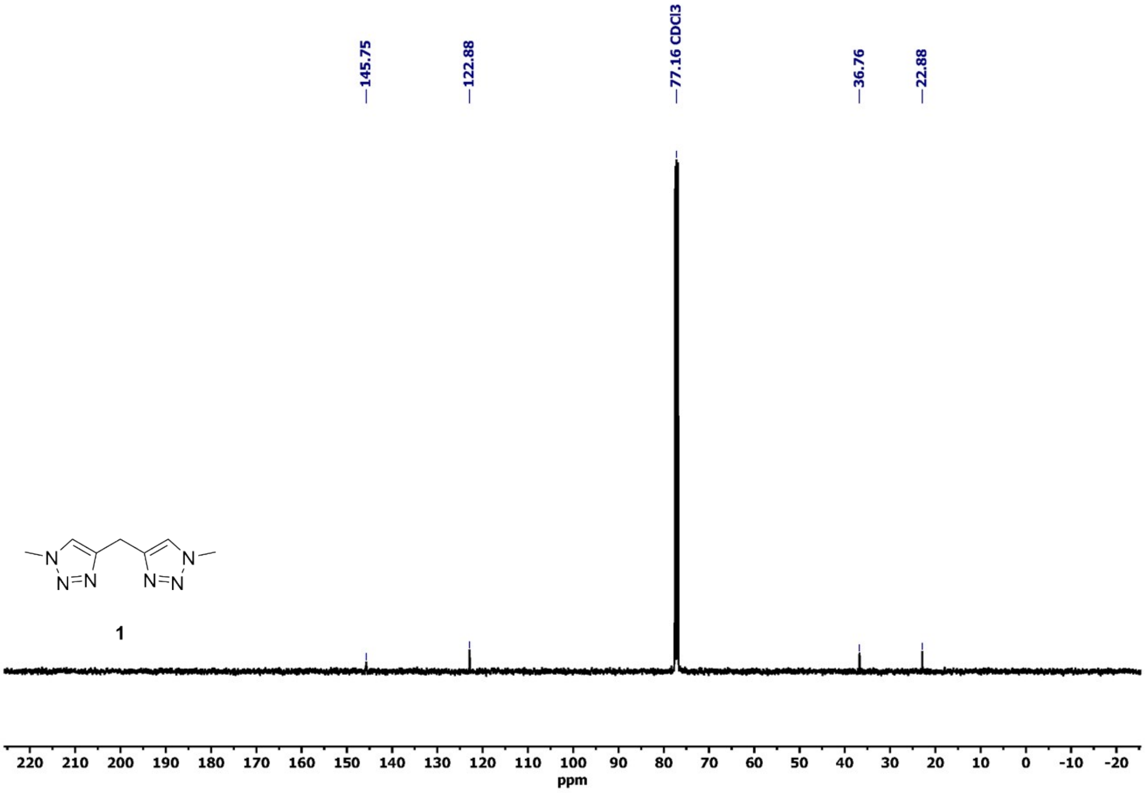

SF

igure S2: *^{13}C NMR spectra of **1** in $CDCl_3$.*

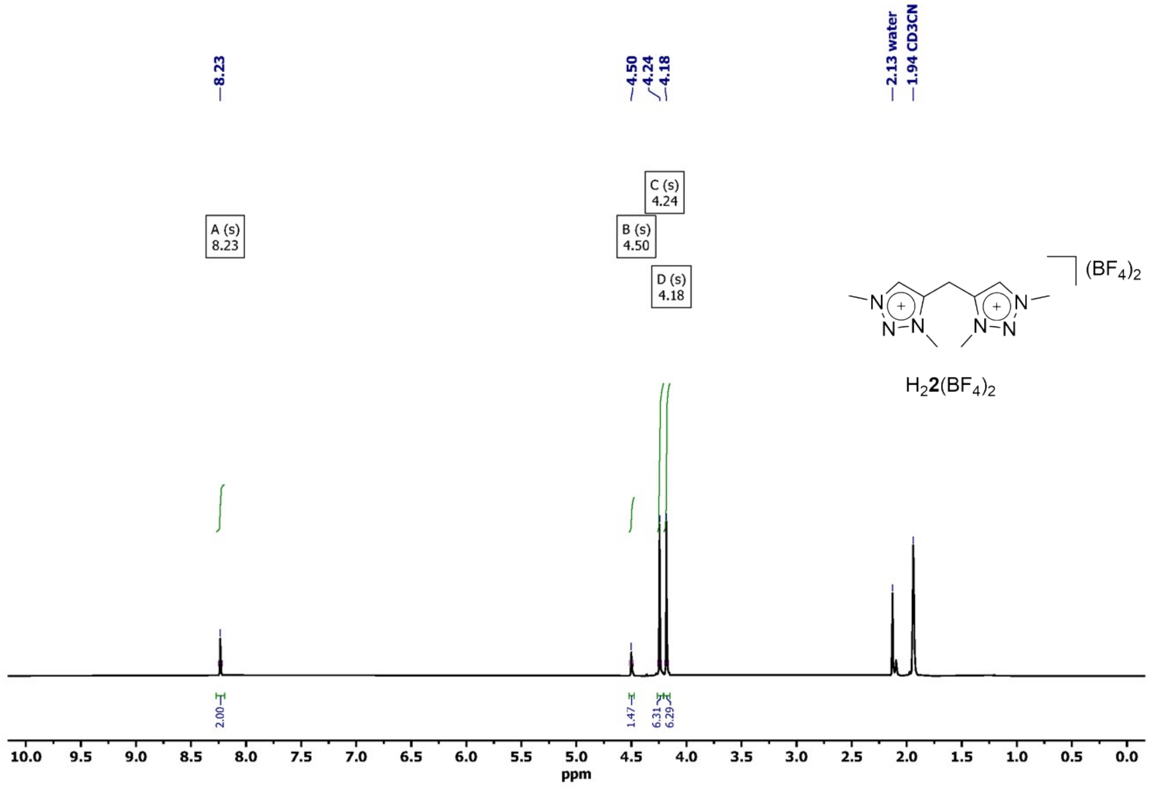

Figure S3: *^{1}H NMR spectra of H_2**2**$(BF_4)_2$ in CD_3CN.*

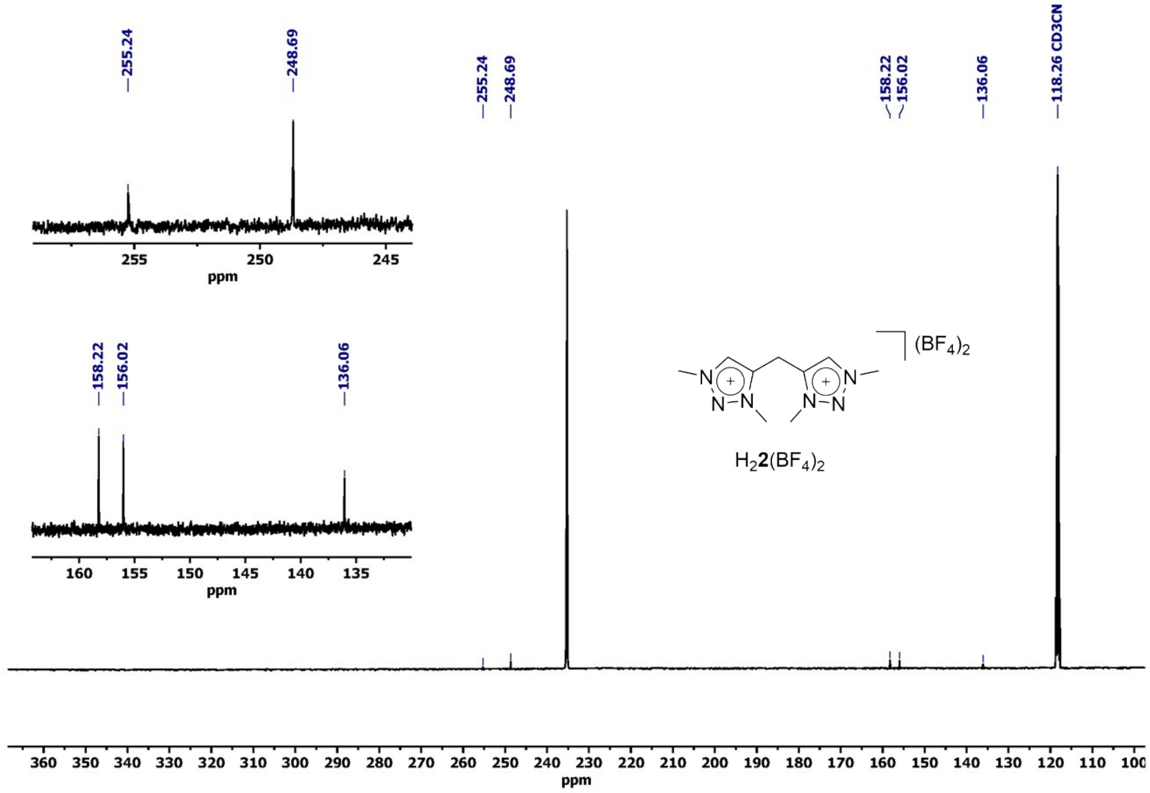

Figure S4: *^{13}C NMR spectra of H_2**2**$(BF_4)_2$ in CD_3CN.*

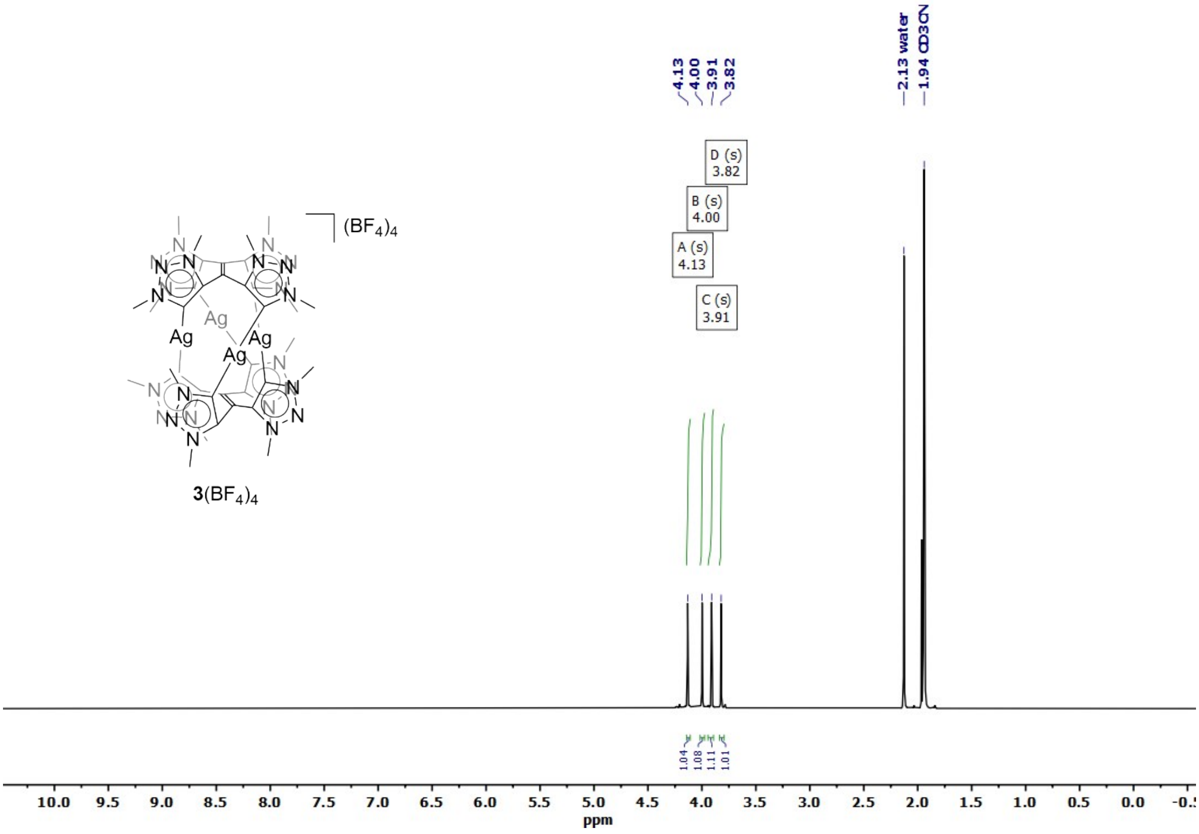

Figure S5: *^{1}H NMR spectra of **3**$(BF_4)_4$ in CD_3CN.*

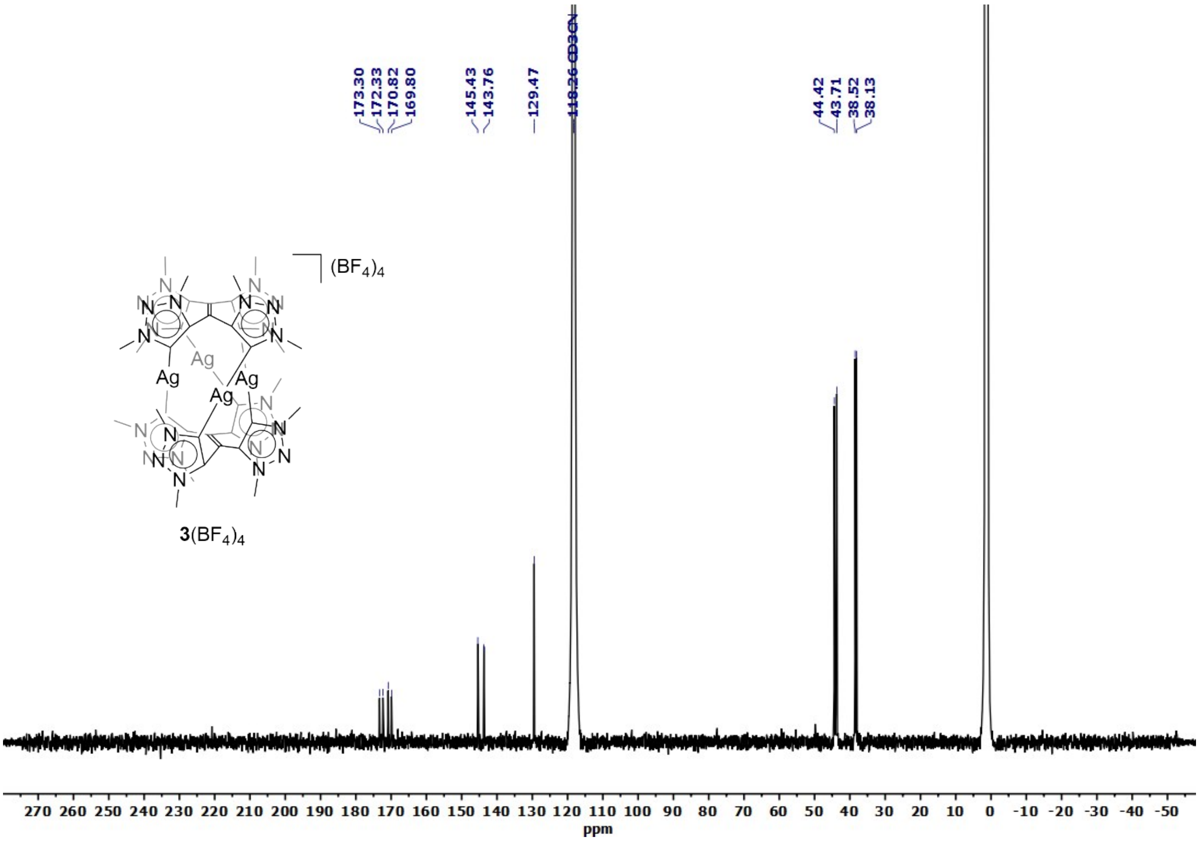

Figure S6: *^{13}C NMR spectra of **3**$(BF_4)_4$ in CD_3CN.*

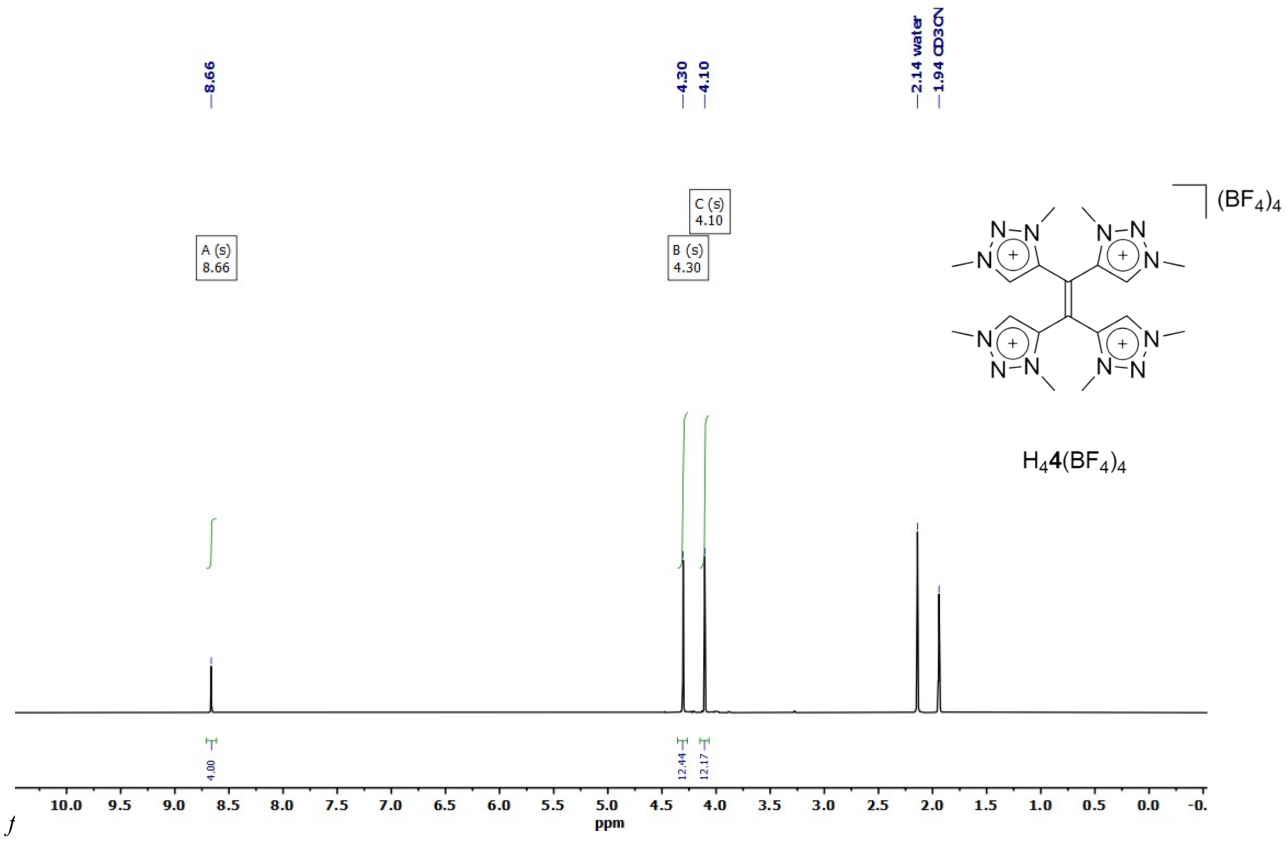

Figure S7: *^{1}H NMR spectra of H_4**4**$(BF_4)_4$ in CD_3CN.*

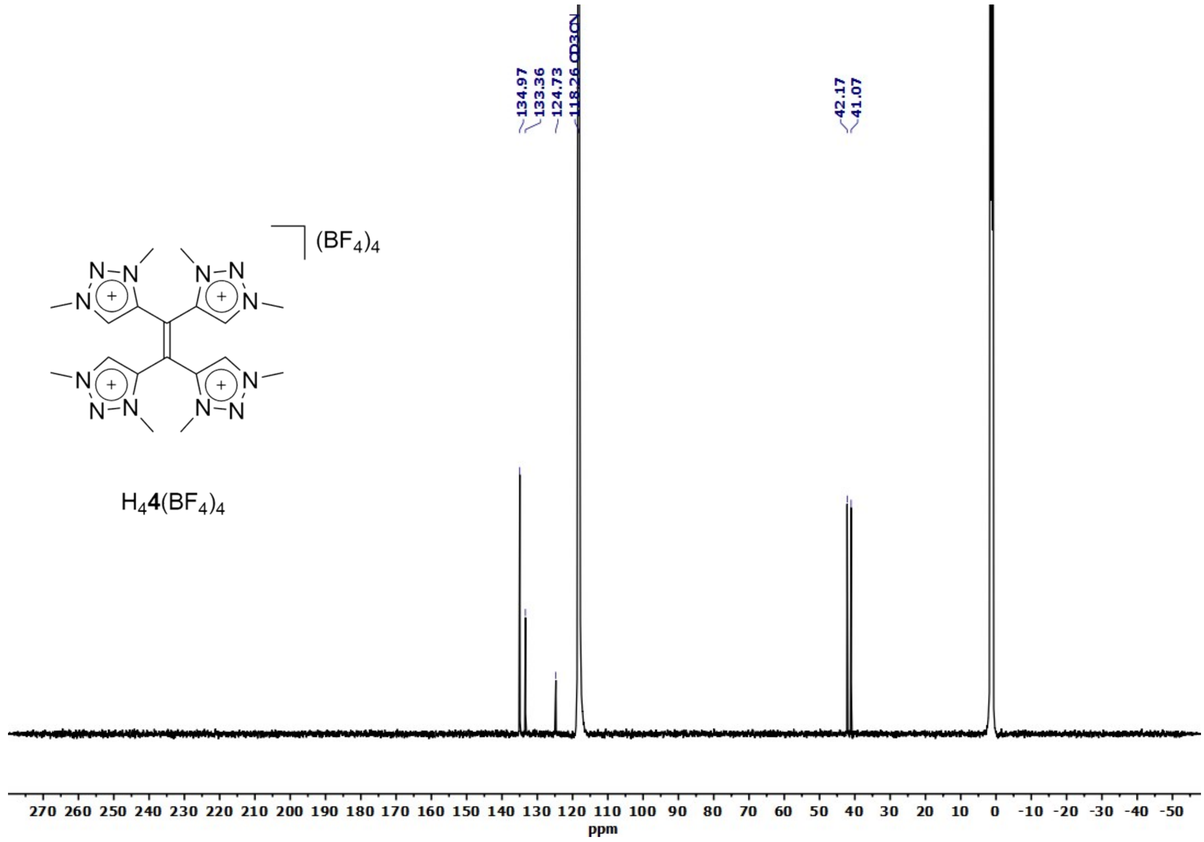

Figure S8: *^{13}C NMR spectra of H_4**4**$(BF_4)_4$ in CD_3CN.*

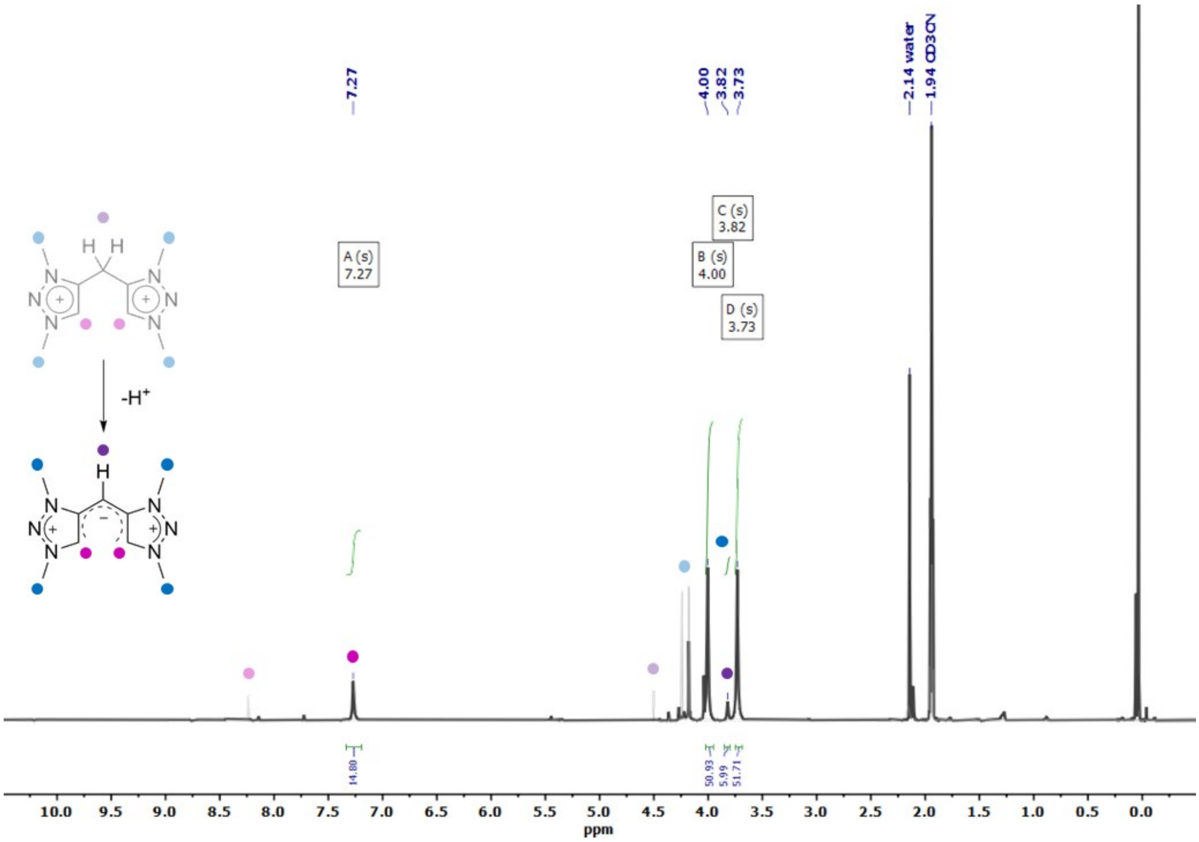

Figure S9: *Grey: ^{1}H NMR spectra of H_2**2**$(BF_4)_2$ in CD_3CN (grey) and black: Crude ^{1}H NMR spectra of $[H_2$**2**$^{2+}](BF_4)$ after deprotonation with KHMDS.*

2. Cyclic Voltammetry

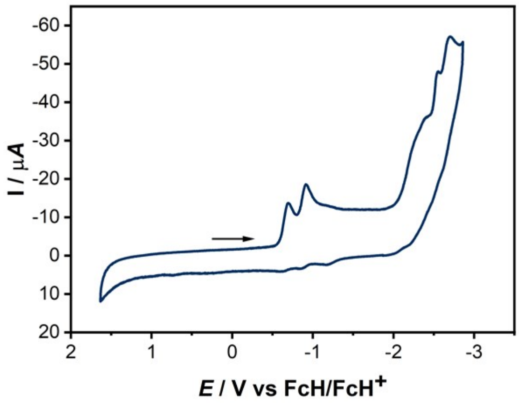

Figure S10: *Cyclic Voltammogram of a 0.1 mM solution of* $H_4\mathbf{4}(BF_4)_4$ *with 0.1 M* NBu_4PF_6 *in acetonitrile at 100 mV·s*$^{-1}$.

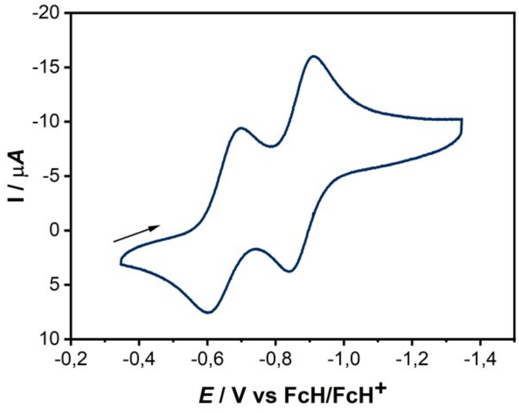

Figure S11: *Cyclic Voltammogram of a 0.1 mM solution of* $H_4\mathbf{4}(BF_4)_4$ *with 0.1 M* NBu_4PF_6 *in acetonitrile at 100 mV·s*$^{-1}$.

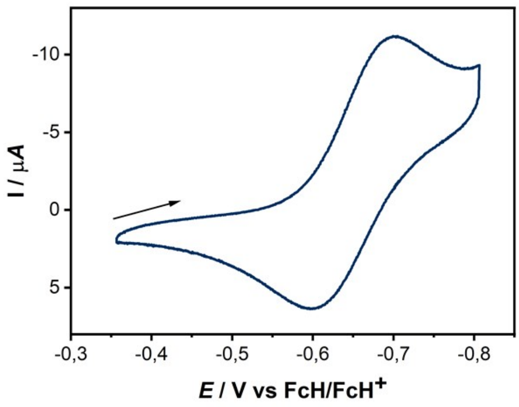

Figure S12 *Cyclic Voltammogram of a 0.1 mM solution of* $H_4\mathbf{4}(BF_4)_4$ *with 0.1 M* NBu_4PF_6 *in acetonitrile at 100 mV·s*$^{-1}$.

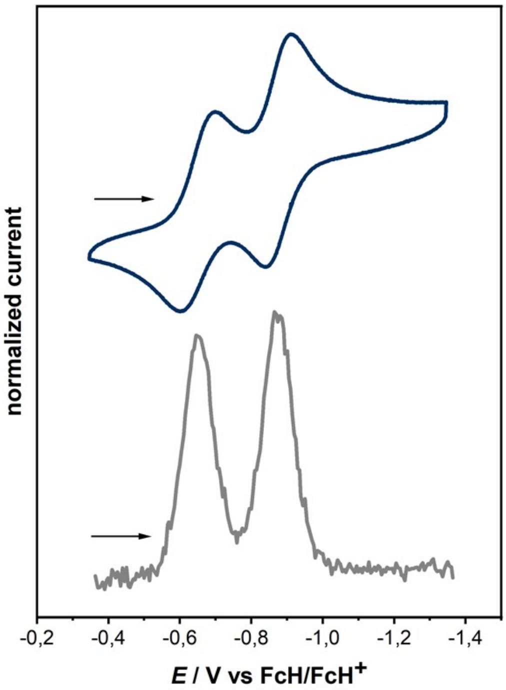

Figure S13: *Top: Cyclic Voltammogram (top) and differential pulse voltammogram (bottom) of a 0.1 mм solution of H_4**4**$(BF_4)_4$ with 0.1 м NBu_4PF_6 in acetonitrile at 100 mV·s^{-1} (top) and 20 mV·s^{-1} (bottom).*

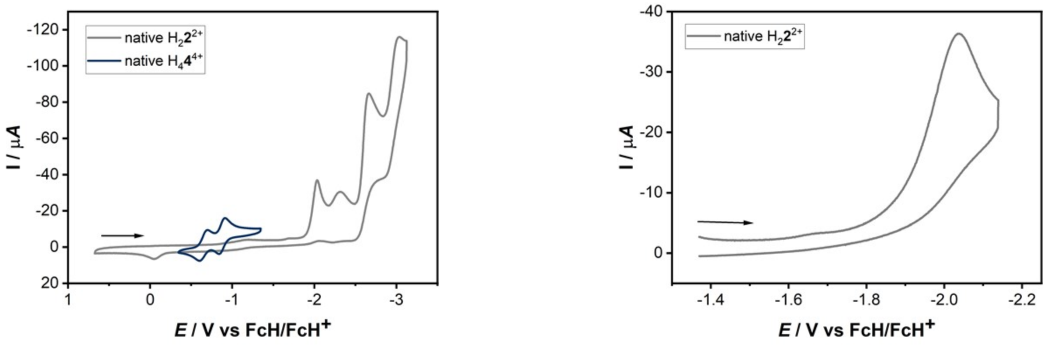

Figure S14 *Combined cyclic voltammograms of a 0.1 mм solution of H_2**2**$(BF_4)_2$ and H_4**4**$(BF_4)_4$ (left) and first reduction of H_2**2**$(BF_4)_2$ (right) with 0.1 м NBu_4PF_6 in acetonitrile at 100 mV·s^{-1}.*

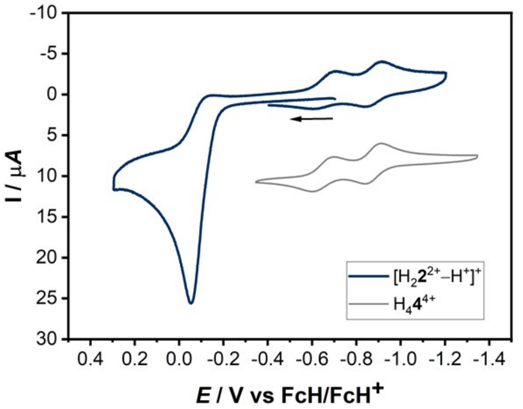

Figure S15 *Combined cyclic voltammograms of a 0.1 mм solution of [H_2**2**$^{2+}$–H^+]$^+$ and H_4**4**$^{4+}$ with 0.1 м NBu_4PF_6 in acetonitrile at 100 mV·s^{-1}.*

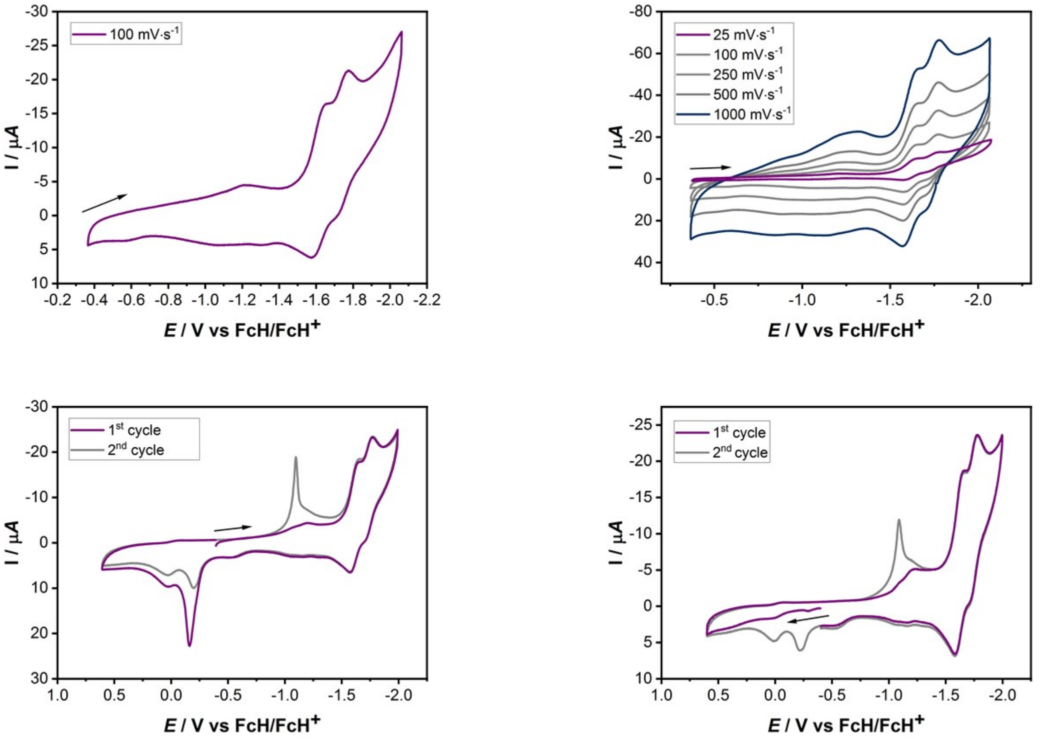

Figure S16: *Cyclic Voltammograms of a 0.1 mм solution of 3(BF_4)$_4$ with 0.1 м NBu_4PF_6 in acetonitrile at different scan rates.*

3. Spectroelectrochemistry

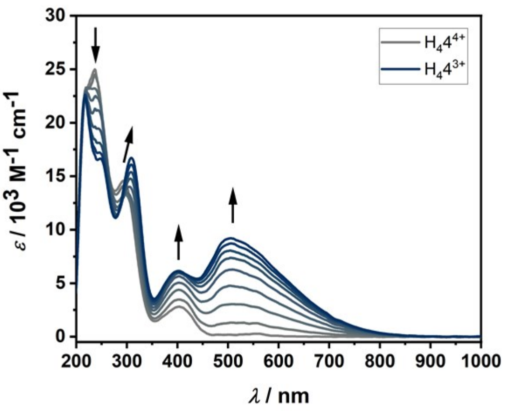

Figure S17: *Changes in UV/vis spectrum of H_4**4**$^{4+}$ in MeCN with 0.1 м NBu_4PF_6 during the first reversible reduction.*

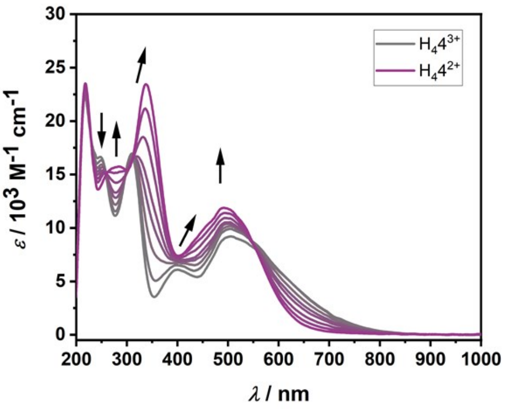

Figure S18: *Changes in UV/vis spectrum of H_4**4**$^{4+}$ in MeCN with 0.1 м NBu_4PF_6 during the second reversible reduction.*

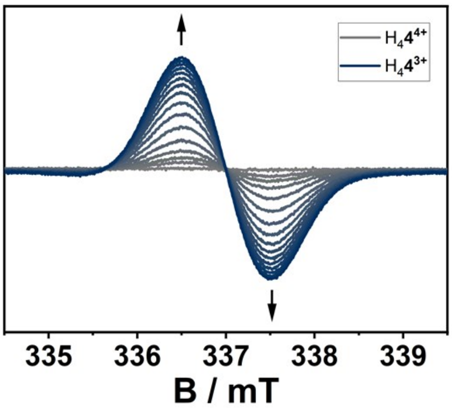

Figure S19: *EPR-spectroelectrochemical measurement of H_4**4**$^{4+}$ with 0.1 м NBu_4PF_6 in acetonitrile, electrolysis at 0.2 V versus silver wire at 0 °C.*

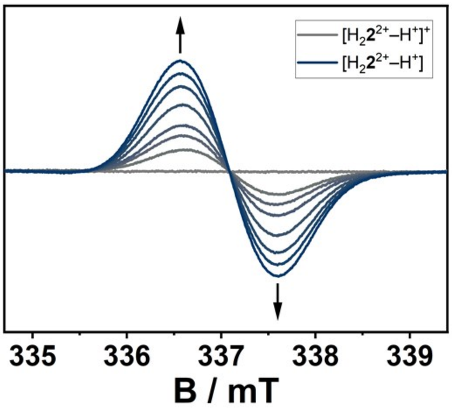

Figure S20: *EPR-spectroelectrochemical measurement of [H_2**2**$^{2+}$–H^+]$^+$ with 0.1 м NBu_4PF_6 in acetonitrile, electrolysis at 0.8 V versus silver wire at 0 °C.*

4. EPR-monitored treatment of $[H_4\mathbf{4}]^{4+}$ with Tetrakis(dimethylamino)ethylene (TDAE)

3.2 mg of $[H_4\mathbf{4}](BF_4)_4$ (4.2 µmol) were dissolved in 2 ml of dry and degassed acetonitrile. A 0.01 M stock solution of TDAE in dry and degassed acetonitrile was prepared. As TDAE can serve as a two-electron reductant,[31] only 0.5 equivalents of TDAE were used to ensure the formation of open-shell species. After combining 0.5 ml (1 µmol) of $[H_4\mathbf{4}](BF_4)_4$ solution and 50 µl (0.5 µmol) of TDAE solution, the resultant bright yellow solution was investigated using EPR spectroscopy at 0 °C. The spectrum shows the characteristic line-rich spectrum of the TDAE radical cation, indicating an electron transfer process. Considering the air-sensitive nature of TDAE, we checked the stock solution EPR spectroscopically under the same experimental conditions. While a small amount of radical was also present in the stock solution, the overlay of both spectra (figure S21) clearly shows that the intense spectrum results from a reaction between TDAE and $[H_4\mathbf{4}]^{4+}$.

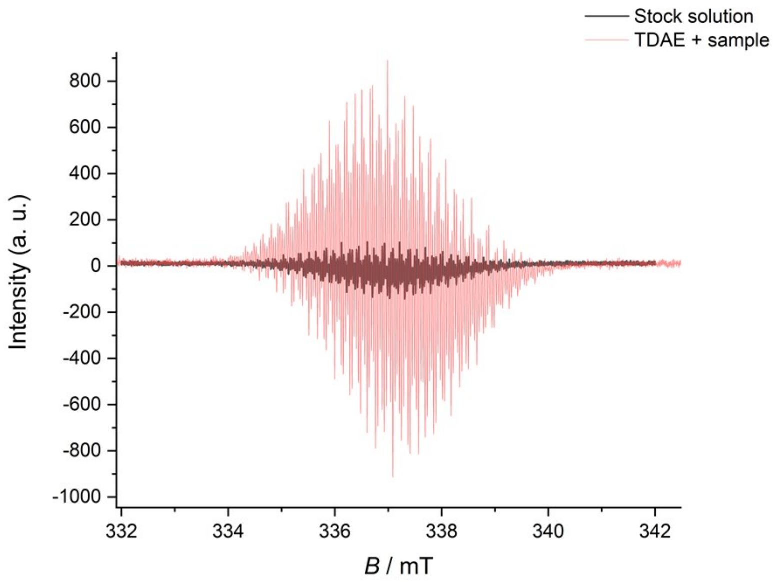

Figure S21: *EPR obtained from reaction between TDAE and $[H_4\mathbf{H}]^{4+}$ (red) and TDAE stock solution (black) at 0 °C.*

5. DFT

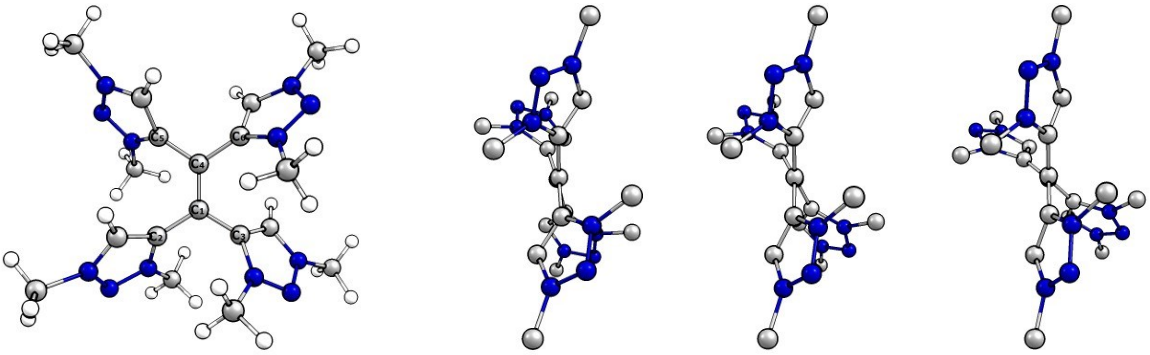

Figure S22: *Left: Optimized structure of native* ***[$H_4$4]$^{4+}$****. Right: Side view along central C–C bond of native, singly and doubly reduced species. H atoms omitted for clarity.*

Table S1: *Selected bond distances and dihedral angles of the various redox forms of* ***[$H_4$4]$^{4+}$****.*

	Native	Singly reduced	Doubly reduced
r(C1–C4) [Å]	1.382	1.438	1.479
r(C1–C2) [Å]	1.478	1.449	1.422
d(C2–C1–C4–C6) [°]	167	152	132

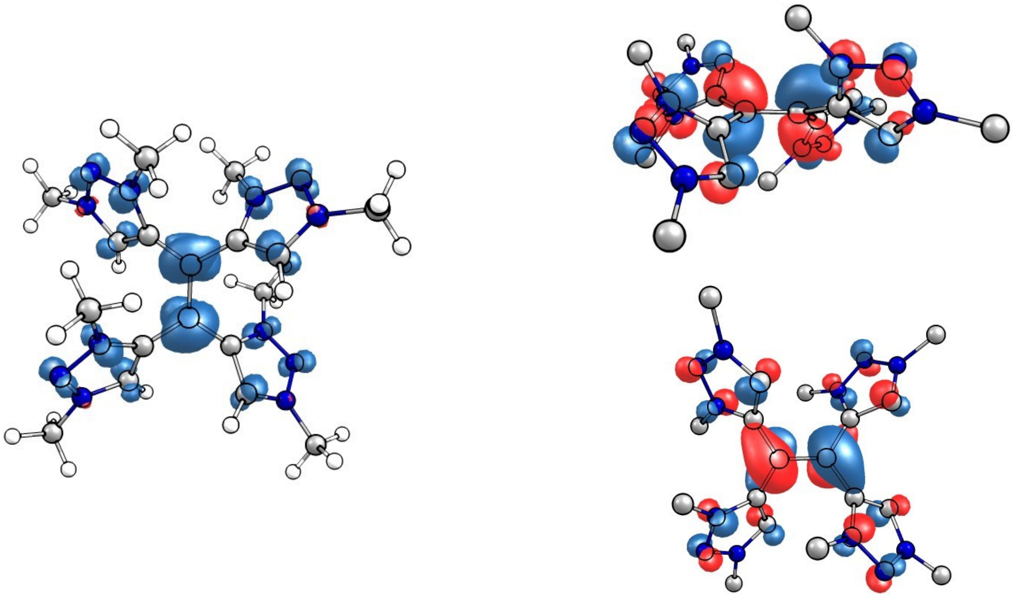

Figure S23: *Left: Spin density of the singly reduced species of* ***[$H_4$4]$^{4+}$*** *(contour value 0.005 $Å^{-1}$). Right: Side and top view of the SOMO of the singly reduced species (contour value 0.05 $Å^{-1}$).*

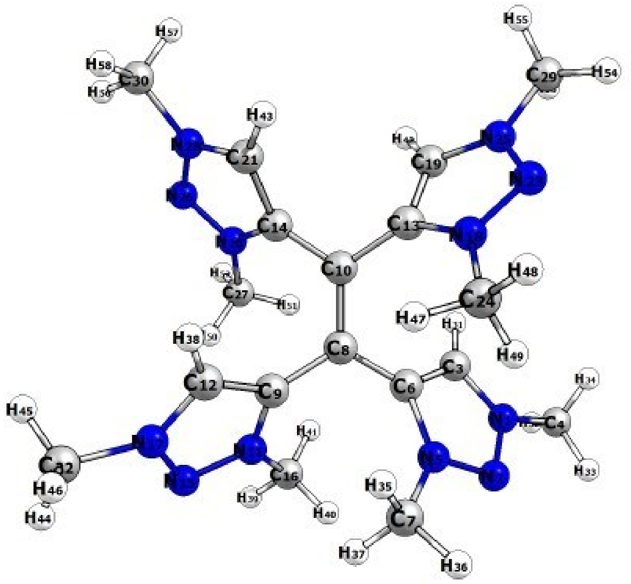

Figure S24: *Structure of $[H_4\mathbf{4}]^{3+\cdot}$ with numbering scheme.*

Table S2: *Spin population and EPR parameters calculated with different functionals.*

	TPSSh	TPSS	B3LYP	M06-2X
Spin population on central C_1–C_4 moiety	40.84 %	39.24 %	41.19 %	43.26 %
g_{iso}	2.0027446	2.0027452	2.002801	2.0029357
A_{N1} (MHz)	-1.755	-1.3136	-2.1043	-3.7469
A_{N2} (MHz)	1.8204	1.5405	2.156	3.3829
A_{N5} (MHz)	3.3774	2.9176	3.9391	5.9646
A_{N11} (MHz)	3.3781	2.9201	3.9381	5.9506
A_{N15} (MHz)	1.8261	1.5478	2.161	3.3867
A_{N17} (MHz)	-1.7515	-1.3118	-2.0993	-3.7387
A_{N18} (MHz)	5.8471	5.216	6.3733	8.6625
A_{N23} (MHz)	1.5035	1.2849	1.8263	2.9022
A_{N25} (MHz)	-1.5002	-1.1251	-1.799	-3.1775
A_{N20} (MHz)	5.8541	5.224	6.3794	8.6679
A_{N26} (MHz)	1.4873	1.2716	1.8066	2.8719
A_{N28} (MHz)	-1.4927	-1.12	-1.789	-3.1556
A_{H31} (MHz)	-5.7691	-5.0889	-5.6669	-5.2235

A_{H38} (MHz)	-5.7603	-5.081	-5.6575	-5.2159
A_{H42} (MHz)	-5.0045	-4.3791	-4.8813	-4.61
A_{H43} (MHz)	-4.9902	-4.3675	-4.8664	-4.5905

The calculated EPR parameters (TPSSh) were employed to simulate EPR spectra with different linewidths, using the EasySpin package[3] implemented in MatLab©. Spectra were simulated for a microwave frequency of 9.45 GHz in the range of 334 to 341 mT. The resulting spectra are shown in figure S25. It is apparent that above a linewidth of 0.07 mT, hyperfine coupling is hardly discernible due to the relatively large number of coupling nuclei with low coupling constants. The spectra with stronger line broadening are very similar to the spectra obtained experimentally during EPR-SEC (see figure S19). While in ideal circumstances very fine linewidths can be obtained, a spectroelectrochemical set-up invariably leads to a certain line broadening (e. g. higher radical concentration at the electrode surface and possible magnetic field inhomogeneity due to conductive Pt wire).

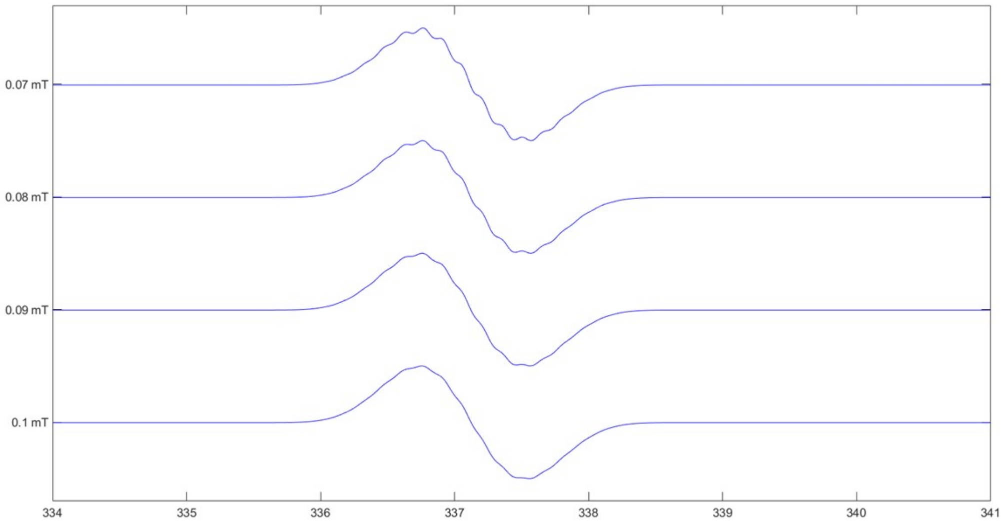

Figure S25: *Linewidth dependence of simulated EPR spectra using the parameters obtained via DFT calculations on the TPSSh/def2-TZVPP level.*

Coordinates of optimized structures:

H_4**4**$^{4+}$:

N	6.163720000000	7.341371000000	8.302839000000
N	6.503120000000	6.676465000000	9.394659000000
C	5.169051000000	6.714234000000	7.615896000000
C	6.878771000000	8.594856000000	7.969259000000
N	5.734010000000	5.600304000000	9.414254000000
C	4.869172000000	5.559574000000	8.330400000000
C	5.892162000000	4.675231000000	10.550563000000
C	3.934843000000	4.455644000000	8.023710000000
C	4.472305000000	3.083833000000	8.146816000000
C	2.627823000000	4.693279000000	7.642573000000
N	5.706816000000	2.660975000000	7.676200000000
C	3.961599000000	1.969316000000	8.803048000000
C	1.962858000000	6.009990000000	7.772798000000
C	1.743390000000	3.657756000000	7.060652000000
N	5.954484000000	1.401493000000	7.996308000000
C	6.725647000000	3.370909000000	6.882456000000
N	4.901366000000	0.991025000000	8.683145000000
N	1.871141000000	6.785874000000	8.917169000000
C	1.218266000000	6.710826000000	6.829049000000
N	2.015660000000	2.849874000000	5.968414000000
C	0.436213000000	3.329257000000	7.406341000000
C	4.879039000000	-0.391858000000	9.213361000000
N	1.153087000000	7.878862000000	8.718975000000
C	2.373487000000	6.542823000000	10.276460000000
N	0.769756000000	7.832874000000	7.453974000000
N	0.990552000000	2.073749000000	5.657945000000
C	3.199241000000	2.788310000000	5.099731000000
N	0.043539000000	2.360597000000	6.535527000000
C	-0.074554000000	8.921890000000	6.911416000000
C	-1.257696000000	1.658149000000	6.454714000000
H	4.780835000000	7.100711000000	6.679086000000

H	7.457095000000	8.438415000000	7.049905000000
H	7.546645000000	8.823293000000	8.806056000000
H	6.144142000000	9.396599000000	7.831250000000
H	4.906307000000	4.334398000000	10.881885000000
H	6.389405000000	5.227577000000	11.355539000000
H	6.522746000000	3.826571000000	10.257614000000
H	3.041152000000	1.822038000000	9.358712000000
H	7.357945000000	2.612347000000	6.407990000000
H	7.346465000000	3.992690000000	7.539451000000
H	6.232055000000	3.979984000000	6.118738000000
H	0.995800000000	6.499449000000	5.787274000000
H	-0.213733000000	3.698546000000	8.194167000000
H	5.836185000000	-0.856347000000	8.955435000000
H	4.049774000000	-0.938073000000	8.747823000000
H	4.752130000000	-0.350722000000	10.302028000000
H	2.429126000000	5.462609000000	10.444071000000
H	1.666337000000	6.993462000000	10.981776000000
H	3.354970000000	7.015680000000	10.401288000000
H	3.887586000000	2.015405000000	5.462009000000
H	3.678958000000	3.772044000000	5.086466000000
H	2.862330000000	2.524175000000	4.091121000000
H	0.406303000000	9.329662000000	6.014383000000
H	-0.161287000000	9.688273000000	7.687975000000
H	-1.062546000000	8.512596000000	6.665901000000
H	-1.187272000000	0.929597000000	5.640979000000
H	-2.042871000000	2.394575000000	6.243337000000
H	-1.449466000000	1.155491000000	7.410306000000

Final Gibbs free energy: -1358.42222994 E_h

H_4**4**$^{3+}$

N	6.076333000000	7.410005000000	7.957994000000
N	6.592695000000	6.801245000000	9.009390000000

C	5.051171000000	6.712527000000	7.400326000000
C	6.664066000000	8.684708000000	7.512786000000
N	5.877862000000	5.683183000000	9.143491000000
C	4.901896000000	5.547880000000	8.158487000000
C	6.153975000000	4.833190000000	10.303188000000
C	3.967004000000	4.449478000000	8.034135000000
C	4.411489000000	3.100112000000	8.313036000000
C	2.607060000000	4.696857000000	7.637159000000
N	5.651079000000	2.561608000000	7.974758000000
C	3.755583000000	2.028913000000	8.925982000000
C	1.923920000000	5.950600000000	7.908921000000
C	1.803251000000	3.713036000000	6.930928000000
N	5.782046000000	1.290951000000	8.358455000000
C	6.750467000000	3.160832000000	7.215966000000
N	4.628655000000	0.986694000000	8.923927000000
N	1.976643000000	6.695965000000	9.080464000000
C	1.058198000000	6.692061000000	7.099948000000
N	2.214186000000	2.886528000000	5.892185000000
C	0.458111000000	3.370959000000	7.095313000000
C	4.441464000000	-0.364273000000	9.479251000000
N	1.239027000000	7.803092000000	9.021775000000
C	2.640395000000	6.391720000000	10.346488000000
N	0.685823000000	7.779993000000	7.822880000000
N	1.242842000000	2.089459000000	5.451377000000
C	3.496102000000	2.841994000000	5.191741000000
N	0.188681000000	2.401256000000	6.183118000000
C	-0.224406000000	8.869791000000	7.432587000000
C	-1.085087000000	1.703979000000	5.938543000000
H	4.535287000000	7.051875000000	6.510144000000
H	7.133342000000	8.541390000000	6.532222000000
H	7.414755000000	8.977535000000	8.252470000000
H	5.873165000000	9.440450000000	7.450657000000
H	5.204474000000	4.468696000000	10.709424000000

H	6.674867000000	5.444948000000	11.046291000000
H	6.791172000000	3.984166000000	10.027611000000
H	2.774286000000	1.962633000000	9.380275000000
H	7.349609000000	2.344983000000	6.799778000000
H	7.385876000000	3.778501000000	7.862138000000
H	6.332012000000	3.773821000000	6.410609000000
H	0.715117000000	6.524886000000	6.085299000000
H	-0.287517000000	3.733310000000	7.793829000000
H	5.370695000000	-0.918124000000	9.317471000000
H	3.610568000000	-0.854218000000	8.958744000000
H	4.227377000000	-0.284941000000	10.551347000000
H	2.697693000000	5.303913000000	10.452583000000
H	2.037993000000	6.819035000000	11.154341000000
H	3.642485000000	6.835667000000	10.369045000000
H	4.155831000000	2.095306000000	5.648892000000
H	3.948897000000	3.837292000000	5.238786000000
H	3.302431000000	2.563598000000	4.151015000000
H	0.119806000000	9.303749000000	6.487097000000
H	-0.202600000000	9.619129000000	8.228987000000
H	-1.237657000000	8.466275000000	7.319732000000
H	-0.904020000000	0.946247000000	5.170901000000
H	-1.828027000000	2.429762000000	5.587464000000
H	-1.422471000000	1.234301000000	6.869466000000

Final Gibbs free energy: -1358.95440599 E_h

H_4**4**$^{2+}$

N	6.038526000000	7.383534000000	7.516167000000
N	6.698743000000	6.869004000000	8.534735000000
C	4.975439000000	6.636338000000	7.124201000000
C	6.532313000000	8.624845000000	6.914704000000
N	6.024066000000	5.738095000000	8.824361000000
C	4.943118000000	5.505962000000	7.961335000000

C	6.344042000000	5.052847000000	10.068323000000
C	4.001105000000	4.443254000000	8.044253000000
C	4.355618000000	3.145619000000	8.506971000000
C	2.602336000000	4.696832000000	7.634720000000
N	5.583484000000	2.494438000000	8.318622000000
C	3.577038000000	2.151015000000	9.126714000000
C	1.871375000000	5.836669000000	8.095867000000
C	1.897558000000	3.817363000000	6.753859000000
N	5.597314000000	1.238950000000	8.810014000000
C	6.707594000000	2.917831000000	7.496113000000
N	4.377591000000	1.065776000000	9.279652000000
N	2.035833000000	6.510178000000	9.306056000000
C	0.867921000000	6.598583000000	7.466581000000
N	2.441266000000	3.014961000000	5.750968000000
C	0.530181000000	3.486350000000	6.689741000000
C	4.049443000000	-0.205818000000	9.929139000000
N	1.248322000000	7.593619000000	9.429187000000
C	2.853132000000	6.143157000000	10.450448000000
N	0.548714000000	7.609106000000	8.312422000000
N	1.532891000000	2.239966000000	5.131648000000
C	3.802457000000	2.987505000000	5.242309000000
N	0.392565000000	2.558947000000	5.710201000000
C	-0.443489000000	8.668706000000	8.114607000000
C	-0.836964000000	1.902598000000	5.258738000000
H	4.338712000000	6.919251000000	6.296246000000
H	6.952397000000	8.411101000000	5.924715000000
H	7.307620000000	9.026987000000	7.572534000000
H	5.702245000000	9.334432000000	6.826362000000
H	5.405817000000	4.720483000000	10.527975000000
H	6.859493000000	5.763396000000	10.721678000000
H	6.986741000000	4.178971000000	9.909565000000
H	2.550717000000	2.170140000000	9.469198000000
H	7.216492000000	2.020197000000	7.131686000000

H	7.422578000000	3.535013000000	8.052906000000
H	6.315255000000	3.503920000000	6.656757000000
H	0.412992000000	6.499718000000	6.489254000000
H	-0.307314000000	3.820206000000	7.288949000000
H	4.878654000000	-0.894960000000	9.747387000000
H	3.124101000000	-0.600878000000	9.495463000000
H	3.922399000000	-0.045499000000	11.006333000000
H	2.838386000000	5.053044000000	10.557050000000
H	2.416599000000	6.616006000000	11.335532000000
H	3.883439000000	6.493648000000	10.321386000000
H	4.437043000000	2.344337000000	5.862429000000
H	4.196276000000	4.009784000000	5.236841000000
H	3.767743000000	2.590206000000	4.223271000000
H	-0.252037000000	9.171930000000	7.160223000000
H	-0.342246000000	9.375565000000	8.942653000000
H	-1.448505000000	8.230550000000	8.114369000000
H	-0.554471000000	1.131886000000	4.536417000000
H	-1.490810000000	2.643022000000	4.783079000000
H	-1.343867000000	1.449847000000	6.118306000000

Final Gibbs free energy: -1359.35399846 E_h

6. Crystal Structures & crystallographic data

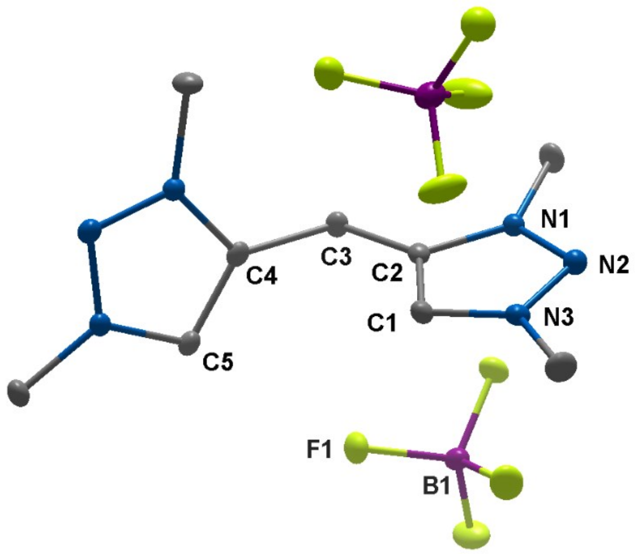

Figure S26: *ORTEP representation of H_2**2**$(BF_4)_2$: ellipsoids drawn at 50% probability. Solvent molecules and H-atoms omitted for clarity.*

Table S3: *Selected bond length and bond angles of H_2**2**$(BF_4)_2$.*

Atom	Bond length (Å)	Atom	Bond angle (°)
C2–C3	1.496(2)	N3–N2–N1	103.9(1)
C3–C4	1.494(2)	N2–N3–C1	112.9(1)
C4–C5	1.371(2)	N3–C1–C2	105.4(1)
C1–C2	1.366(2)	N1–C2–C1	105.2(1)
N1–C2	1.354(2)	N2–N1–C2	112.6(1)
N1–N2	1.323(2)	C2–C3–C4	112.5(1)
N2–N3	1.318(2)	C1–C2–C3	132.5(1)
C1–N1	1.349(2)	C5–C4–C3	132.0(1)

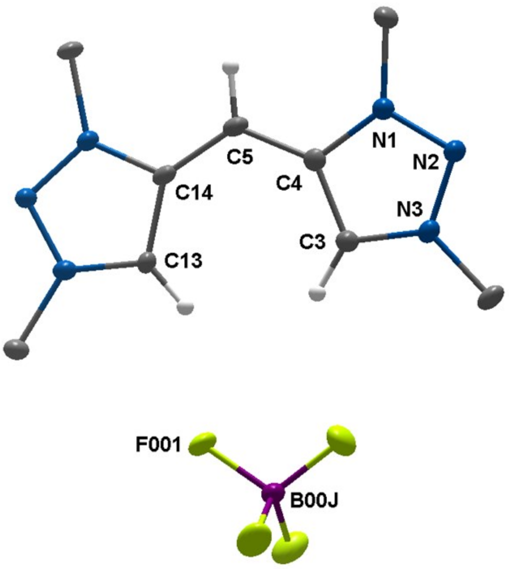

Figure S27: *ORTEP representation of [H_2**2**$^{2+}$–H^+](BF_4) ellipsoids drawn at 50% probability. Solvent molecules and H-atoms omitted for clarity.*

Table S4: *Selected bond length and bond angles of [H_2**2**$^{2+}$–H^+](BF_4).*

Atom	Bond length (Å)	Atom	Bond angle (°)
C4–C5	1.404(5)	N3–N2–N1	102.1(3)
C5–C14	1.388(6)	N2–N3–C3	114.3(3)
C13–C14	1.407(5)	N3–C3–C4	106.3(3)
N1–C4	1.372(5)	N1–C4–C3	103.0(3)
N1–N2	1.354(4)	N2–N1–C4	114.3(3)
N2–N3	1.330(5)	C14–C5–C4	124.2(4)
N3–C3	1.344(5)	C3–C4–C5	135.1(4)
C3–C4	1.401(5)	C5–C14–C13	134.6(4)

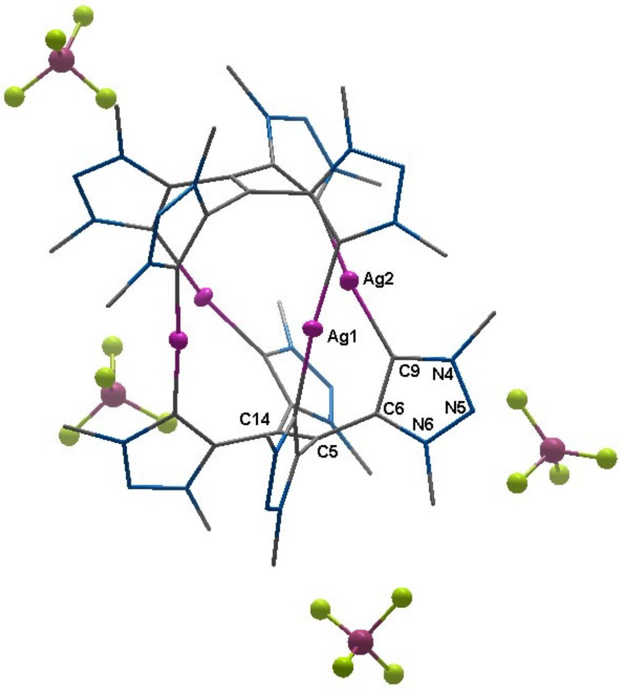

Figure S28: *ORTEP representation of* ***3****$(BF_4)_4$ ellipsoids drawn at 50% probability. Solvent molecules and H-atoms omitted for clarity.*

Table S5: *Selected bond length and bond angles of* ***3****$(BF_4)_4$.*

Atom	Bond length (Å)	Atom	Bond angle (°)
Ag1–Ag2	3.619(1)	C19–Ag1–C1	172.8(2)
Ag2–Ag3	3.531(1)	C28–Ag2–C9	172.1(2)
Ag3–Ag4	3.336(1)	C27–Ag4–C10	176.2(2)
Ag1–Ag4	3.348(1)	C36–Ag3–C18	171.1(2)
C5–C14	1.336(8)	C14–C5–C6	120.8(5)
C5–C6	1.484(7)	C9–C6–C5	129.0(5)
C6–C9	1.383(7)	C6–C9–N4	101.6(4)
C9–N4	1.370(6)	C9–N4–N5	116.0(4)
N4–N5	1.318(6)	N6–N5–N4	102.5(4)
N5–N6	1.313(7)	C6–N6–N5	113.2(4)
N6–C6	1.361(7)	C9–C6–N6	106.8(4)

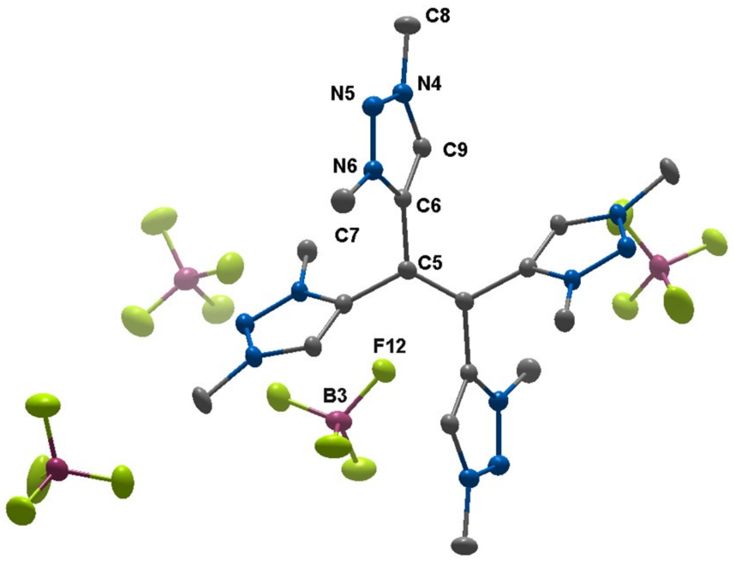

Figure S29: *ORTEP representation of H_4**4**$(BF_4)_4$ ellipsoids drawn at 50% probability. Solvent molecules, counter anions and H-atoms omitted for clarity.*

Table S6: *Selected bond length and bond angles of H_4**4**$(BF_4)_4$.*

Atom	Bond length (Å)	Atom	Bond angle (°)
C5–C5	1.343(5)	C5–C5–C6	122.4(2)
C5–C6	1.486(5)	C4–C5–C6	114.6(2)
C6–C9	1.371(4)	C9–C6–C5	131.6(2)
N4–C9	1.348(4)	C6–C9–N4	105.6(2)
N4–N5	1.321(4)	C9–N4–N5	112.6(2)
N6–N5	1.318(4)	N4–N5–N6	104.6(2)
N6–C6	1.367(3)	N5–N6–C6	112.1(2)
		C9–C6–N6	105.1(2)

	H_2**2**$(BF_4)_2$	$[H_2$**2**$^{2+}–H^+](BF_4)$	**3**$(BF_4)_4$	H_4**4**$(BF_4)_4$
Chemical formula	$C_9H_{16}B_2F_8N_6$	$C_9H_{15}B_1F_4N_6$	$C_{42}H_{57}Ag_4B_4$ $F^{16}N_{27}$	$C_{22}H_{34}B_4F_{16}N_{14}$
M_r	381.90	294.08	1718.59	841.88
Crystal system	Triclinic	Monoclinic	Triclinic	Triclinic
Space group	$P\bar{1}$	C2/*m*	$P\bar{1}$	$P\bar{1}$
a (Å)	7.1669(8)	10.5350(8)	11.052(3)	10.15(2)
b (Å)	9.743(2)	6.4885(5)	14.637(4)	11.83(3)
c (Å)	11.689(2)	18.717(2)	21.244(5)	15.93(4)
α (°)	90.304(4)	90	85.032(7)	97.92(9)
β (°)	91.835(6)	92.323(2)	81.796(8)	92.65(7)
γ (°)	105.407(4)	90	77.855(7)	102.91(7)
V ($Å^3$)	786.3(2)	1278.4(2)	3319.5(14)	1841(8)
Z	2	4	2	2
Densitiy (g cm^{-3})	1.613	1.528	1.7192	1.5186
F(000)	388	608	1693.5905	856.6978
Radiation Type	$MoK_α$	$MoK_α$	$MoK_α$	$MoK_α$
μ (mm^{-1})	0.167	0.139	1.261	0.152
Crystal size	0.24x0.23x0.08	0.21x0.17x0.02	0.18x0.15x0.09	0.28x0.07x0.04
Meas. Refl.	13141	1452	64288	34133
Indep. Refl.	3208	1452	15858	7514
Obsvd. [$I > 2σ(I)$] refl.	2720	1193	11831	5771
R_{int}	0.0287	0.0739	0.0695	0.0475
R [$F^2 > 2σ(F^2)$],	0.0327, 0.0827,	0.0628, 0.1569,	0.0571, 0.1146,	0.0465, 0.1360,
wR(F^2), S	1.018	1.574	1.0432	1.1245
$Δρ_{max}$, $Δρ_{min}$ (e $Å^{-3}$)	0.250, –0.315	0.650, –0.678	2.0808, –1.7892	0.7968, –0.5748
CCDC number	2025037	2025018	2025039	2025043

7. References

[1] G. R. Fulmer, A. J. M. Miller, N. H. Sherden, H. E. Gottlieb, A. Nudelman, B. M. Stoltz, J. E. Bercaw, K. I. Goldberg, *Organometallics* **2010**, *29*, 2176–2179.

[2] M. Krejčik, M. Daněk, F. Hartl, *J. Electroanal. Chem. Interfacial Electrochem.* **1991**, *317*, 179–187.

[3] S. Stoll, A. Schweiger, *J. Magn. Reson.* **2006**, *178*, 42–55.

[4] F. Neese, *WIREs Comput Mol Sci* **2012**, *2*, 73–78.

[5] F. Neese, *WIREs Comput Mol Sci* **2018**, *8*, 1327–1332.

[6] A. D. Becke, *Phys. Rev. A* **1988**, *38*, 3098–3100.

[7] F. Weigend, R. Ahlrichs, *Phys. Chem. Chem. Phys.* **2005**, *7*, 3297–3305.

[8] S. Grimme, *J. Comput. Chem.* **2006**, *27*, 1787–1799.

[9] S. Grimme, *J. Comput. Chem.* **2004**, *25*, 1463–1473.

[10] S. Grimme, J. Antony, S. Ehrlich, H. Krieg, *J. Chem. Phy.* **2010**, *132*, 154104.

[11] S. Grimme, S. Ehrlich, L. Goerigk, *J. Comput. Chem.* **2011**, *32*, 1456–1465.

[12] J. Tao, J. P. Perdew, V. N. Staroverov, G. E. Scuseria, *Phys. Rev. Lett.* **2003**, *91*, 146401.

[13] P. J. Stephens, F. J. Devlin, C. F. Chabalowski, M. J. Frisch, *J. Phys. Chem.* **1994**, *98*, 11623–11627.

[14] Y. Zhao, D. G. Truhlar, *Theor. Chem. Acc.* **2007**, *120*, 215–241.

[15] R. Izsak, F. Neese, *J. Chem. Phy.* **2011**, *135*, 144105.

[16] J. L. Whitten, *J. Chem. Phy.* **1973**, *58*, 4496–4501.

[17] O. Vahtras, J. Almlöf, M. W. Feyereisen, *Chem. Phys. Lett.* **1993**, *213*, 514–518.

[18] F. Neese, F. Wennmohs, A. Hansen, U. Becker, *Chem. Phys.* **2009**, *356*, 98–109.

[19] F. Neese, *J. Comput. Chem.* **2003**, *24*, 1740–1747.

[20] K. Eichkorn, O. Treutler, H. Öhm, M. Häser, R. Ahlrichs, *Chem. Phys. Lett.* **1995**, *240*, 283–289.

[21] F. Weigend, *Phys. Chem. Chem. Phys.* **2006**, *8*, 1057–1065.

[22] K. Eichkorn, F. Weigend, O. Treutler, R. Ahlrichs, *Theor. Chem. Acc.* **1997**, *97*, 119–124.

[23] A. L. Spek, *J. Appl. Crystallogr.* **2003**, *36*, 7–13.

[24] G. M. Sheldrick, *SHELXS-97, Program for Crystal Structure Solution and Refinement*, University of Göttingen, Göttingen, Germany, **1997**.

[25] G. M. Sheldrick, *SADABS Ver. 2008/1, SADABS. Program for Empirical Absorption Correction*, University of Gottingen, Germany, **2012**.

[26] G. M. Sheldrick, *Acta Crystallogr., Sect. C: Struct. Chem.* **2015**, *71*, 3–8.

[27] G. M. Sheldrick, *SHELXL Version 2014/7, Program for Chrystal Structure Solution and Refinement*, University of Göttingen, Germany, **2014**.

[28] G. M. Sheldrick, *Acta Crystallogr., Sect. A: Found. Crystallogr.* **2008**, *64*, 112–122.

[29] R. Maity, A. Mekic, M. van der Meer, A. Verma, B. Sarkar, *Chem. Comm.* **2015**, *51*, 15106–15109.

[30] L. Zhang, R. Das, C. T. Li, Y. Y. Wang, F. E. Hahn, K. Hua, L. Y. Sun, Y. F. Han, *Angew. Chem. Int. Ed.* **2019**, *58*, 13360–13364.

[31] J. Broggi, T. Terme, P. Vanelle, *Angew. Chem. Int. Ed.* **2014**, *53*, 384–413.
